# The microbial community from the early-plant colonizer (*Baccharis linearis*) is required for plant establishment on copper mine tailings

**DOI:** 10.1038/s41598-021-89769-1

**Published:** 2021-05-17

**Authors:** María Consuelo Gazitúa, Verónica Morgante, María Josefina Poupin, Thomas Ledger, Gustavo Rodríguez-Valdecantos, Catalina Herrera, María del Carmen González-Chávez, Rosanna Ginocchio, Bernardo González

**Affiliations:** 1 Laboratorio de Bioingeniería, Facultad de Ingeniería y Ciencias, Universidad Adolfo Ibáñez, 7941169 Santiago, Chile; 2 Desarrollo e Innovación (PIDi), Programa Institucional de Fomento a la Investigación, Universidad Tecnológica Metropolitana, Santiago, Chile; 3 Centro de Investigación en Recursos Naturales y Sustentabilidad (CIRENYS), Universidad Bernardo O’Higgins, Santiago, Chile; 4 Center of Applied Ecology and Sustainability (CAPES), Santiago, Chile; 5 Programa de Edafología, Colegio de Postgraduados en Ciencias Agrícolas, Montecillo, México; 6 Pontificia Universidad Católica de Chile, Facultad de Agronomía, Santiago, Chile; 7 Present Address: Viromica Consulting, Santiago, Chile

**Keywords:** Microbiology, Plant sciences

## Abstract

Plants must deal with harsh environmental conditions when colonizing abandoned copper mine tailings. We hypothesized that the presence of a native microbial community can improve the colonization of the pioneer plant, *Baccharis linearis*, in soils from copper mining tailings. Plant growth and microbial community compositions and dynamics were determined in cultivation pots containing material from two abandoned copper mining tailings (Huana and Tambillos) and compared with pots containing fresh tailings or surrounding agricultural soil. Controls without plants or using irradiated microbe-free substrates, were also performed. Results indicated that bacteria (*Actinobacteria*, *Gammaproteobacteria,* and *Firmicutes* groups) and fungi (*Glomus* genus) are associated with *B. linearis* and may support plant acclimation, since growth parameters decreased in both irradiated (transiently without microbial community) and fresh tailing substrates (with a significantly different microbial community). Consistently, the composition of the bacterial community from abandoned copper mining tailings was more impacted by plant establishment than by differences in the physicochemical properties of the substrates. Bacteria located at *B. linearis* rhizoplane were clearly the most distinct bacterial community compared with those of fresh tailings, surrounding soil and non-rhizosphere abandoned tailings substrates. Beta diversity analyses showed that the rhizoplane bacterial community changed mainly through species replacement (turnover) than species loss (nestedness). In contrast, location/geographical conditions were more relevant than interaction with the plants, to explain fungal community differences.

## Introduction

Copper mining operations adversely affect the environment due to the deposition of large volumes of hard-rock waste materials in nearby areas, being mine tailings quite relevant sources of contamination^1^. Mine tailings are waste materials resulting from the mineral separation process, which are mostly composed of silt or sand-sized particles. They lack organic matter and nutrients while containing high quantities of heavy metals^2^ and slightly alkaline to low pH^3,4^. Abandoned mining tailings may also result in secondary environmental impacts due to the dispersal of metal-rich particles and weathering of the material with the generation of acid mine drainage^3^. These characteristics of tailings limit microbial diversity^5^, as well as spontaneous plant colonization^6^ and, therefore, affect the application of phytoremediation procedures.

Conventional remediation technologies based on chemical/physical treatments are generally nonviable economic options in post-operative mining tailings generated by large size mine operations^6^. Application of organic amendments (alone or in combination with beneficial microorganisms, see below) to degraded soils has been successful in some cases^7,8^. In addition, plant-based technologies are generally cost-effective and environmentally sustainable^6^. Phytoremediation technologies allow metal immobilization, reduce metal contamination in the surrounding environments, and provide erosion control and a wildlife habitat^9^. In particular, the use of native plants in these technologies is also favored because they demonstrate tolerance to local environmental conditions and provide a foundation for natural ecological successions^1,10^. Spontaneous primary succession involves plants and, although initially limited to small patches of vegetation distributed mainly on their edges^11^, in the long-term, it leads to vegetation changes and transformation of mineral substrates into the soil^12^. The role that microorganisms have in growth, nutrition and health in plants is increasingly better known. Among other capacities, microorganisms play a key role in plant establishment as they degrade organic matter and recycle nutrients^13^ and protect plants from stress^14,15^. However, the extent of the influence of microbial community structure, and especially microbial diversity, on colonizer plant growth on copper tailings has not been approached so far^5,16^. The role of native microbial communities in plant establishment in these degraded substrates is less understood. In this work, we investigated both the native microbial community structure and diversity and their contribution to early plant colonizers’ development in soils from copper mining tailings. We used a pioneer plant *Baccharis linearis* as a study model, and substrates originated from two abandoned copper mining tailings from a semiarid zone in the central part of Northern Chile (Huana and Tambillos). We first analyzed the physicochemical properties of these tailing substrates. Then, we analyzed their microbial communities by means of terminal restriction fragment length polymorphism (T-RFLP) and clone library analyses. Comparison with those found in a surrounding agricultural soil and contrasting different compartments according to their association with plants (non-rhizosphere, rhizosphere, and rhizoplane), was also carried out. Subsequently, we studied the effects of fresh tailings in the establishment and growth of *B. linearis* plants, and, finally, using gamma-irradiation we studied the effect of a reduction in the microbial community in the germination, growth, and establishment of this pioneer plant.

## Results

Copper mining tailings having different ages and therefore showing different *B. linearis* successional levels were compared among them. A recently produced copper mining tailing (fresh tailing), and a surrounding soil from Tambillos mine chosen as control soil (Table 1) were also analyzed. When appropriate, root compartments (non-rhizosphere, rhizosphere and rhizoplane) were also contrasted. Firstly, the physicochemical properties of each copper mining tailing substrates were analyzed. Subsequently, a microbiological characterization was performed by culture-dependent endophytic and rhizospheric microbial cell counts, and by culture-independent approach T-RFLP profiling and clone library. 16S rRNA sequences and internal transcribed spacer (ITS) sequences were used for bacterial and fungal communities’ analysis, respectively. Finally, the effects of the microbial community on the germination of *B. linearis* seeds and growth of its seedlings on these substrates were also determined. The latter two experimental settings differed in the developmental stage where *B. linearis* was exposed to these substrates. For some conditions, the substrates were previously irradiated to substantively decrease the presence of microorganisms. Foliar area, dry biomass, primary root length, number of secondary roots, photosynthetic pigment content and germination rate were measured.

**Table 1:** Main description of studied sites.

Sites	Localization (altitude)	Climate	Sample type	Soil type^a^	Mining operation/ closure date/ operative status	Vegetation (spontaneous colonization by pioneer plants)^b^
Tambillos (Coquimbo)	30° 12′ 06" S 71° 14′ 27" W (204 m.a.s.l)	Coastal steppe Semi-arid	Soil (waste materials resulting from the extraction of copper and gold)	Technosols^a^	Copper and gold 1987 Abandoned mining tailings	High
Huana (Ovalle)	30° 42′ 24" S 70° 57′ 20" W (454 m.a.s.l)	Dessert	Soil (waste materials resulting from the extraction of copper)	Technosols	Copper 1998 Abandoned mining tailings	Moderate
Tambillos fresh tailing (Coquimbo)	30° 12′ 06" S 71° 14′ 27" W (204 m.a.s.l)	Coastal steppe Semi-arid	Soil (waste materials resulting from the extraction of copper and gold)	Technosols	Copper and ore – Active mine operation	No
Surrounding soil (Ovalle)	30° 42′ 24" S 70° 57′ 20" W (454 m.a.s.l.)	Dessert	Non-perturbed soil in the area of Huana mine	Aridisols	No mining activities	High

^a^Also classified as Thionic-Technosols due to their high levels of sulphidic materials.

^b^Two of the most abundant native species found in both tailings were *Baccharis linearis* (Romerillo, 19% cover) and *Haplopappus parvifolius* (Bailahuén, 1% cover)^17^. Neither forestation nor direct vegetation managements have been applied in the sites.

### Physico-chemical characterization of substrates

In order to explore the most relevant challenges faced by plants colonizing tailings and their rhizosphere microbiota, a physicochemical characterization of rhizosphere and non-rhizosphere substrates of copper mining tailings was performed. Two tailings, Huana and Tambillos, displaying a medium and a low level of colonization by *B. linearis*, respectively (Table 1), were considered for study. These copper mining tailings are located in the Coquimbo Region in Northern-Central Chile, which has a semiarid Mediterranean climate. Tambillos tailings used for sampling was closed in 1983, approximately, and its material comes from copper/gold flotation processes. Huana tailings has materials coming from copper flotation processes and was abandoned in 1998. Along with these substrates, characterization of the rhizosphere and non-rhizosphere of a control soil (from the vicinity of Huana tailings) was initially performed. Some of the parameters analyzed were also compared to a freshly produced tailing substrate from the Tambillos site (fresh tailings) (Table 1). Determination of pH in water extracts showed that all tailing substrates were neutral to slightly alkaline (7.14 to 7.95) (Table 2). Rhizosphere substrates showed a higher pH than non-rhizosphere substrates, a trend also observed in surrounding soil samples, where the rhizosphere compartment reached pH up to 8.35. Cation exchange capacity, as well as the dissolved organic carbon content of rhizosphere substrates, were also higher than those of non-rhizosphere counterparts in all locations under study, reaching the highest value in the rhizosphere of surrounding soil. Electrical conductivity values in the rhizosphere substrates were lower than in their corresponding non-rhizosphere substrates. However, the values observed in tailing samples exceeded those favorable for plant growth^18^, being especially high in non-rhizosphere tailing substrates and fresh Tambillos tailings (Table 2).

**Table 2:** Physicochemical characterization of rhizosphere and non-rhizosphere substrates from mine tailings and surrounding soil.

Substrate parameters ^†,¶^	Substrates						
	Tambillos tailings		Huana tailings		Surrounding soil		Fresh tailings
	R	N-R	R	N-R	R	N-R	N-R
Soil pH soil:H_2_O 1:1	7.89 ± 0.19	7.14 ± 1.0	7.95 ± 0.17	7.52 ± 1.46	8.35 ± 0.42	7.09 ± 0.69	7.69 ± 0.15
EC (mS cm^−1^)	2.6 ± 0.7↑	5.2 ± 0.5↑↑	3.5 ± 1.0↑	7.2 ± 2.1↑↑	0.3 ± 0.1	1.7 ± 0.2	4.3 ± 0.3↑↑
CEC (meq 100 g^−1^)	27.0 ± 8.9	8.7 ± 4.9	31.7 ± 6.8	6.7 ± 3.1	35.0 ± 11.4	14.7 ± 5.1	24. 3 ± 7.1
DOC (mg L^−1^)	42.5 ± 17.0	21.5 ± 6.0	25.0 ± 5.0	6.0 ± 2.5	98.0 ± 11.0	34.0 ± 10.5	ND^‡‡^
Soluble SO_4_^2−^ (g L^−1^)	0.52 ± 0.2↑↑	2.97 ± 0.39	0.88 ± 0.49↑↑	1.73 ± 0.26	0.03 ± 0.01	0.32 ± 0.08	0.94 ± 0.28↑↑
**Texture (%)**							
< 2 µm	12.8 ± 3.8	13.6 ± 10	23.1 ± 8.1	10.3 ± 5	15.4 ± 7.8	16.9 ± 6	24.9 ± 11.3
50–2 µm	35.1 ± 4.6	43.6 ± 13	53.0 ± 8.4	36.5 ± 18	14.9 ± 15.1	22.2 ± 9	60.2 ± 23.7
200–50 µm	52.2 ± 6.3	42.8 ± 19	23.9 ± 12.1	53.1 ± 21	69.8 ± 22.9	60.9 ± 11	16.0 ± 29.7
Soil type	Loam	Loam	Silty loam	Sandy loam	Sandy loam	Sandy Loam	Silty loam

*R* rhizosphere, *N-R* non-rhizosphere, *EC* electrical conductivity, *CEC* cation exchange capacity, *DOC* dissolved organic carbon.

^**†**^Values are given as mean and standard error (2–6 replicates).

^**¶**^↑ High, or ↑↑ very high, according to standard analytical soil parameters.

^‡‡^ND: not determined.

Soluble sulfate concentrations were in the very high range in all the analyzed compartments, compared to those of surrounding soil rhizosphere and non-rhizosphere (Table 2). Texture analysis of substrate samples showed a greater proportion of fine-sized particle material (lower than 50 µm) in the rhizosphere of Huana and fresh Tambillos tailings (76.1% and 85.1%, respectively). Those of Tambillos tailings and surrounding soil (47.9% and 30.3%, respectively) (Table 2), were clearly lower. About one-fourth of total material in the rhizosphere of Huana and fresh Tambillos tailings corresponded to particles smaller than 2 µm (23.1% and 24.9%, respectively).

In order to compare the potential of rhizosphere tailing substrates to support plant growth in terms of macronutrient availability and saline concentrations, their nutritional status and main cationic components were determined (Supplementary Table S1 online). Standard analytical soil tests^19^ indicated that organic matter contents were very low in almost all the analyzed substrates. Unexpectedly, organic matter content in fresh tailings was three times higher than in the surrounding soil and in Tambillos tailings, and six times higher than in Huana tailings. Available N and P levels were lower in all tailing substrates relative to the surrounding soil rhizosphere, with the level of available P in Huana tailings rhizosphere being extremely low (Supplementary Table S1 online).

### Root-associated microbial community in *B. linearis* inhabiting abandoned tailings

**Figure 1:**
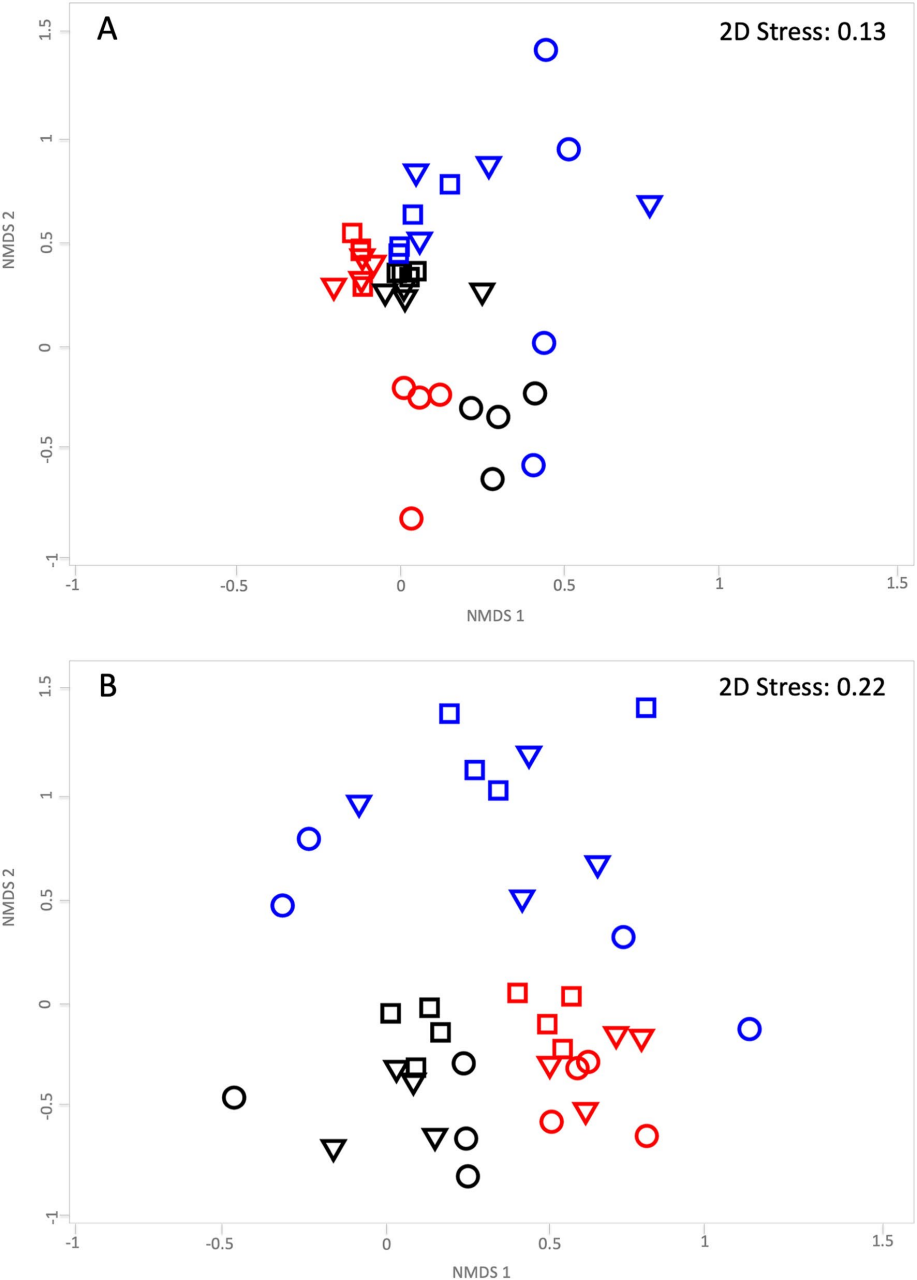
Non-metric multidimensional scaling (NMDS) analysis of *Hha*I-terminal restriction fragment length polymorphisms (T-RFLP) profiles of metagenomic DNA from three soil substrates. T-RFLP profiles of 16S rRNA bacterial **(A)** and internal transcribed spacer fungal **(B)** community sequences were obtained from the rhizoplane (circles), rhizosphere (triangles), and non-rhizosphere (squares) of Huana tailings (red symbols), Tambillos tailings (blue symbols) and surrounding soil (black symbols). Each symbol corresponds to a single T-RFLP profile (four samples each site). Stress values for NMDS analysis are shown in each upper right corner. This image was created using Primer v6 software, URL https://www.primer-e.com/.

The microbial community associated to individuals of *B. linearis* naturally occurring in Huana and Tambillos tailings, as well as the surrounding soil, were characterized. Three compartments: the rhizoplane (substrate adhered to the roots), the rhizosphere and non-rhizosphere substrates, were considered. T-RFLP profiles of the more abundant species in bacterial communities were first compared by non-metric multidimensional scaling (NMDS) analysis of the corresponding samples, using Bray–Curtis distance matrices. Results showed a clear clustering among most rhizoplane samples (Fig. 1A). Two-way ANOSIM comparisons between “root compartment” and “localization” groups indicated that observed grouping (by root compartment) was statistically supported with a global R-value of 0.51 (p = 0.001). This suggested moderate to high differences in the taxonomic composition according to Clarke^20^, i.e. an R-value ranging from 0.5 to 1. These results were also supported by ANOSIM pairwise test. Rhizoplane compartments strongly contributed to detected differences (R-values for rhizoplane/rhizosphere, rhizoplane/non-rhizosphere, and rhizosphere/non-rhizosphere pairs were 0.59, 0.74, and 0.30, respectively). Pairwise test by localization revealed minor differences in bacterial community composition between Huana tailings/surrounding soil, Tambillos tailings/surrounding soil, or Huana tailings/Tambillos tailings pairs (R-values of 0.003, 0.296, and 0.256, respectively).

Alpha diversity indices were estimated using T-RFLP profiles, relative abundances for the dominant operational taxonomic units obtained from Huana and Tambillos tailings, and surrounding soil, and compared by “root compartment” and “localization” as factors (Supplementary Table S2 online). *H´* diversity index values were 2.06 ± 0.61, 2.94 ± 0.49, and 3.20 ± 0.20 for rhizoplane, rhizosphere, and non-rhizosphere, respectively, representing average values of the three locations. ANOVA showed that localization did not significantly affect *H´* (p ≤ 0.2080) whereas statistically significant differences were found among root compartments (p ≤ 0.0001). The relatively reduced diversity observed in rhizoplane surrounding soil was also reflected by evenness (*J´*) and richness (*S´*) indices (Supplementary Table S2 online). *S´* values denoted an increase in the average number of the more abundant, therefore detectable by T-RFLP, putative species, measured as operational taxonomic units, in the bacterial community at rhizosphere and non-rhizosphere compartments compared to that of rhizoplane (p ≤ 0.0001). *S´* and *H´* indices in non-rhizosphere compartments increased with abandonment time (*S´* of 39.50 ± 3.11 and *H´* of 3.33 ± 0.15 for Tambillos tailings vs. *S´* of 32.50 ± 10.24 and *H*´ of 2.97 ± 0.41 for Huana tailings). The lowest evenness values observed at the rhizoplane compartment indicate low similarity in dominant species abundance compared to that in the rhizosphere and non-rhizosphere compartments (p ≤ 0.001).

**Figure 2:**
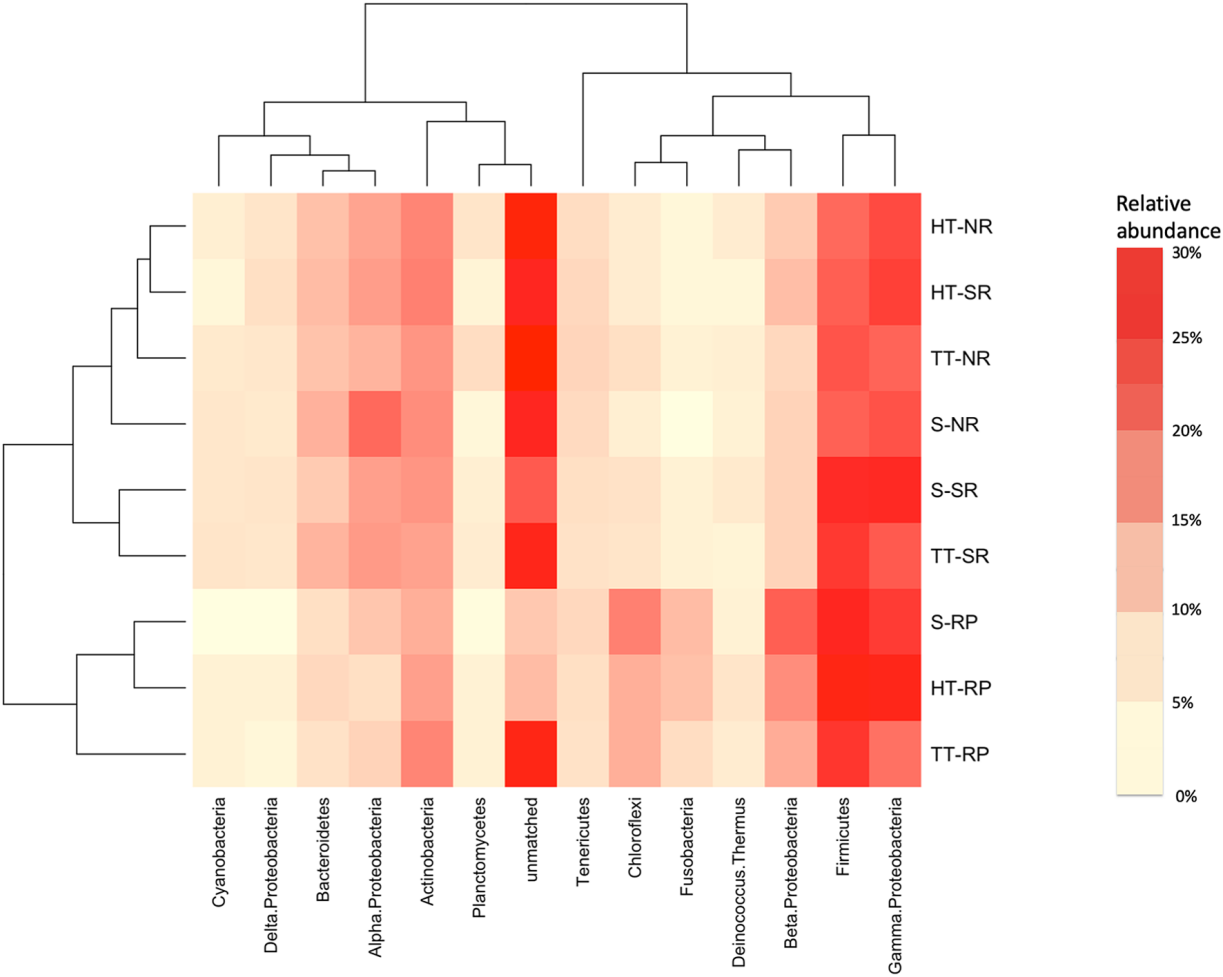
Relative abundance of main bacterial taxa detected by terminal restriction fragment length polymorphisms. Two-way clustering dendrogram obtained using Spearman distance with average grouping algorithm to evaluate the distribution of bacterial communities from the different sites and compartments at the phylum level. The heat map colors represent the relative abundance percentage (right hand legend) of the bacterial phylum (X-axis clustering) within each site/compartment (Y-axis clustering). *HT-NR* Huana tailings-non rhizosphere, *HT-SR* Huana tailings rhizosphere, *TT-NR* Tambillos tailings-non rhizophere, *S-NR* surrounding soil-non rhizosphere, *S-SR* surrounding soil rhizosphere, *TT-SR* Tambillos tailings rhizosphere, *S-RP* surrounding soil rhizoplane, *HT-RP* Huana tailings rhizoplane, *TT-RP* Tambillos tailings rhizoplane. This image was created using RStudio Team. Version 1.0.143. URL http://www.rstudio.com/.

Terminal restriction fragment assignation allowed getting a preliminary idea on bacterial community members. All samples were dominated by *Proteobacteria* (33.61%), most of them affiliated to *Alpha, Beta,* and *Gamma* classes (7.66%, 6.62%, and 17.84%, respectively), followed by *Firmicutes* (18.45%), *Actinobacteria* (10.20%), *Bacteroidetes* (5.01%)*, Chloroflexi* (4.25%)*, Tenericutes* (2.95%) and *Fusobacteria* (2.0%) (Fig. 2)*. Actinobacteria*, *Gammaproteobacteria,* and *Firmicutes* groups were ubiquitous and abundant phyla both in Huana and Tambillos tailings, and in the surrounding soil root compartments. *Chloroflexi, Fusobacteria,* and *Betaproteobacteria* were abundant phyla only in the rhizoplane microhabitat. *Bacteroidetes* and *Alphaproteobacteria* were more abundant in the rhizosphere and non-rhizosphere than in rhizoplane microhabitats. Considering the rhizoplane of *B. linearis* showed a strong similarity in T-RFLP profiles and dominant taxa abundances in all locations (Fig. 2, Supplementary Table S2 online), bacterial communities from this microhabitat were further characterized. A prospective 16S rRNA gene clone library analysis of metagenomic DNA from *B. linearis* rhizoplanes obtained from Huana and Tambillos tailings, and surrounding soil was performed. In agreement with results shown above (Fig. 2), *Proteobacteria* dominated (almost 60% of sequences) this 16S rRNA clone library (Supplementary Fig. S1 online). Almost fifty-two percent of clones affiliated with *Gammaproteobacteria,* mainly belonging to *Pseudomonadaceae* and *Enterobacteriaceae*. Around five percent of clones affiliated with *Betaproteobacteria* (mainly associated to *Oxalobacteriaceae*), while *Alphaproteobacteria* were less represented (2.42%). *Actinobacteria* comprised 20.97% with most sequences affiliating with uncultured strains (> 97% identity).

In contrast to bacterial community, NMDS analysis of T-RFLP profiles of the more abundant fungal species showed a clear grouping according to “localization” (Fig. 1B). Two-way ANOSIM comparisons between “root compartment” and “localization” groups were subsequently performed. A global R-value of 0.71 (p = 0.001) indicated that observed grouping by localization was statistically supported. This was also reinforced by the ANOSIM pairwise test where the “root compartment” factor (rhizoplane, rhizosphere, and non-rhizosphere) showed modest differences in fungal community composition (global R-value of 0.331, p = 0.001). This test also revealed higher differences between rhizoplane/non-rhizosphere pair than for rhizoplane/rhizosphere or rhizosphere/non-rhizosphere pairs (R-value of 0.61, 0.30, and 0.04, respectively).

The H´, *J*´ and *S*´ indices were estimated from relative abundances of the dominant fungal operational taxonomic units obtained for each sample and also compared by “root compartment” and “localization” as factors (Supplementary Table S3 online). One-way ANOVA showed statistically significant differences within localization (p ≤ 0.01 and p ≤ 0.0001 for H´ and S´, respectively). Comparison of *S´* and *H´* values suggested higher complexity of fungal communities in Tambillos tailings. In comparative terms, diversity indices tended to increase with time of abandonment (Tambillos vs. Huana), as observed for bacterial communities. No significant differences in *J’* values were observed when compared localization and root compartments (Supplementary Table S3 online).

**Figure 3:**
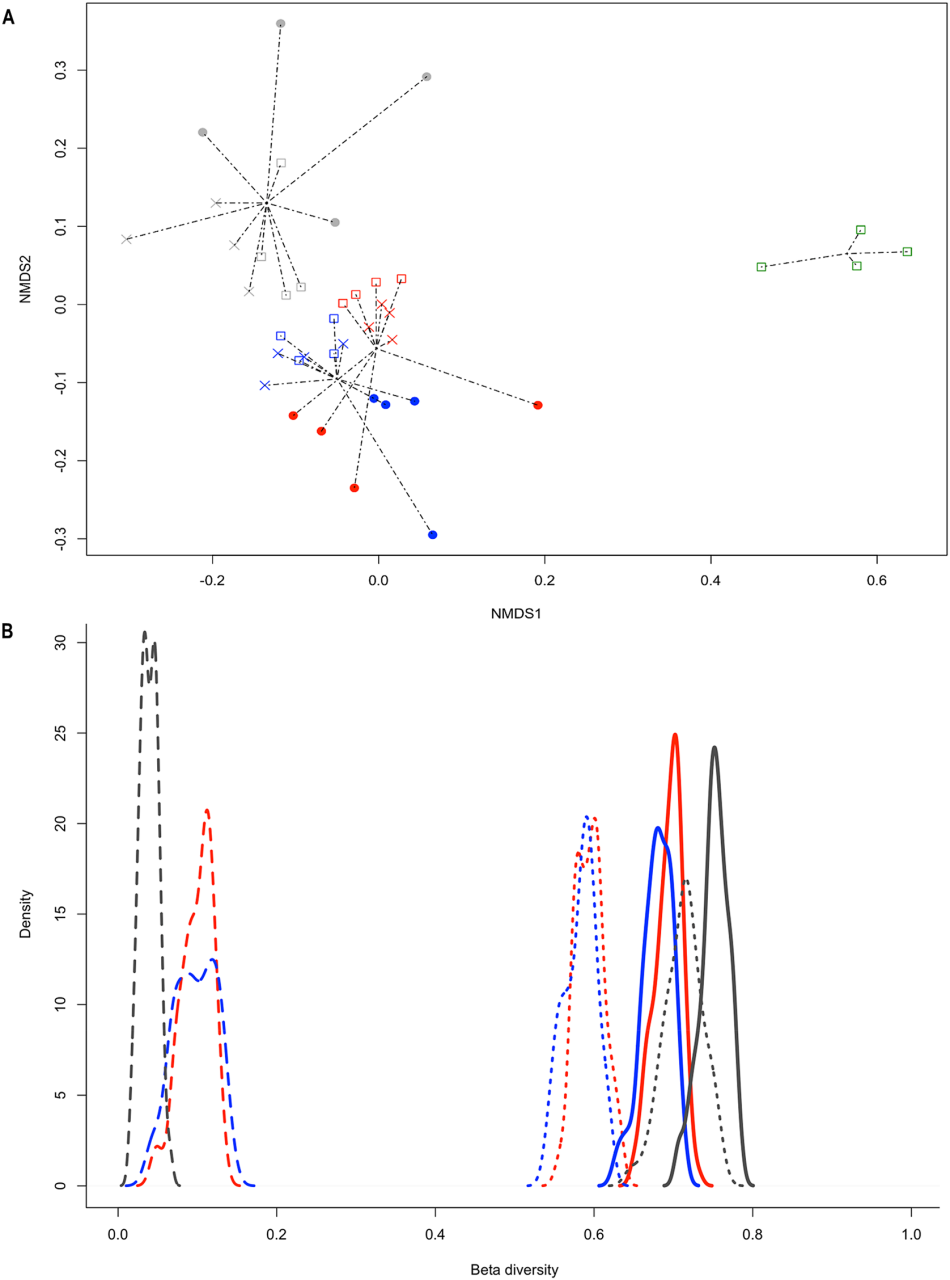
Beta diversity analyses of microbial meta-community of the non-rhizosphere, the rhizosphere and rhizoplane of *Baccharis linearis* plants inhabiting abandoned tailings and surrounding soil. **(A)** Non-metric multidimensional scaling analysis of meta-community dissimilarities. Grouping by localization and root compartments was performed by estimation of the total beta diversity distances (β_SOR_) between samples. The localization and root compartment are denoted as follows: Huana tailings (blue), Tambillos tailings (dark grey), fresh tailings (green), surrounding soil (red), rhizoplane (filled circle), rhizosphere (x) and non-rhizosphere (□). Each point corresponds to a single T-RFLP profile (n = 4 replicates). **(B)** Density plots representing the distribution of the total beta diversity (β_SOR_), turnover (β_SIM_) and nestedness (β_SNE_) arising from multiple-site dissimilarity across the samples. Components of multiple-site dissimilarity were computed for microbial community composition (bacteria and fungi) in Huana (blue), and Tambillos (black) tailings and surrounding soil (red). Β_SNE_: dashed line; β_SIM_: dotted line and β_SOR_: solid line. These figures were created using Betapart R Package Version 1.3. URL http://CRAN.R-project.org/package=betapart.

An additional comparison of abundant members from bacterial and fungal communities associated with *B*. *linearis* roots was accomplished by beta diversity analysis. A strong dissimilarity in profiles from fresh Tambillos tailing samples compared with Huana and Tambillos tailings, and surrounding soil samples was observed (Fig. 3A). This grouping was statistically supported (ANOSIM test performed over β_SOR_ dissimilarity between locations) with a global R-value of 0.558 (p = 0.001), suggesting moderate to high differences in taxonomic composition, according to Clarke^20^. Due to the strong dissimilarity observed in fresh Tambillos tailings microbial community composition, these samples were excluded from the following beta diversity studies. Microbial community composition of Tambillos tailing samples was more dissimilar with respect to the pattern of compositional changes detected between Huana tailings and surrounding soil samples (Fig. 3A). These results were supported by two-way ANOSIM analysis (with localization and root compartment as factors) showing a global R-value of 0.516 (p = 0.001). Pairwise test revealed that rhizoplane was the main root compartment factor explaining observed differences between Tambillos tailings with respect to Huana tailings and surrounding soil samples. The pairwise R-values were 0.622 (p = 0.001) for rhizoplane/rhizosphere pair, 0.716 (p = 0.001) for the rhizoplane/non-rhizosphere, and 0.298 (p = 0.001) for the rhizosphere/non-rhizosphere pair. To further investigate processes driving observed differences in microbial meta-community composition of *B. linearis*, a beta diversity partition analysis was performed. Resulting distributions of β_SOR_, β_SIM_ and β_SNE_ values are shown in Fig. 3B. β_SOR_, β_SIM_ and β_SNE_ dissimilarity showed similar distribution for Huana tailings and surrounding soil meta-community composition profiles. Microbial composition in Tambillos tailings revealed higher total dissimilarity (β_SOR_) levels than Huana tailings and surrounding soil, attributable to the high value of the β_SIM_ component (Fig. 3B).

### Effects of the microbial community on germination and growth of *B. linearis* in copper mine tailings

**Figure 4:**
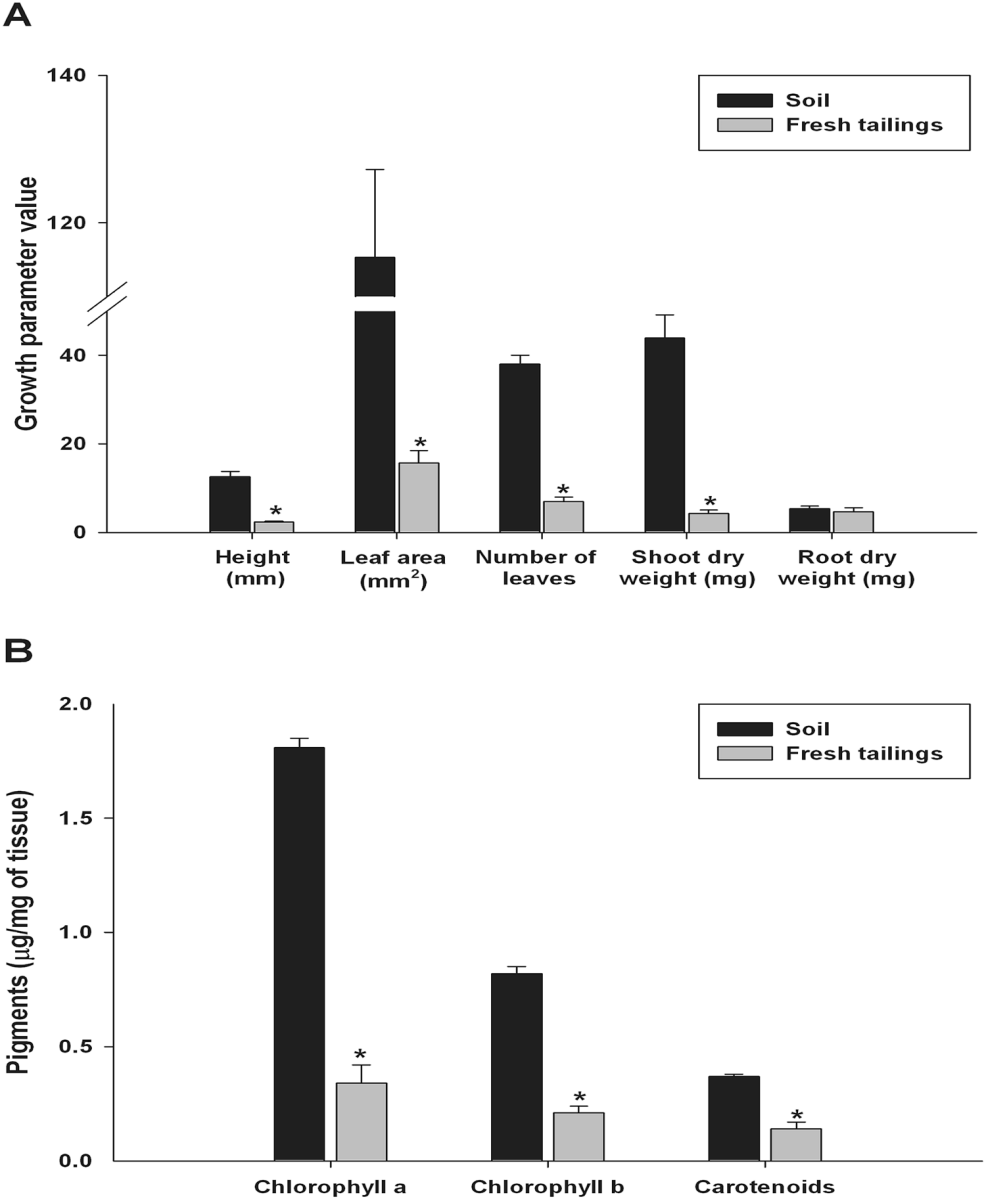
Comparisons of the effects of fresh tailings and a surrounding soil substrate in the growth and the establishment of *Baccharis linearis* plants in tailing substrates. **(A)** Growth parameters and **(B)** pigment contents in 18 weeks old plants that were transferred to a surrounding soil (black bars) or to a substrate derived from Tambillos fresh tailings (grey bars), six weeks after sowing. Bars represent the average ± SE of 24 replicates and asterisks indicate significant differences considering the treatment (surrounding soil or fresh tailings) as factor in each parameter (one-way ANOVA for parametric data or Kruskal–Wallis test for non-parametric data, p < 0.01).

To explore the roles of microbial communities associated with *B*. *linearis* growing on copper mining tailings, the effects of tailings in the establishment of *B. linearis* plants after transplantation were analyzed. In a first approach, seeds were sown in sterile conditions in inert substrate and after six weeks plantlets were transferred to fresh Tambillos tailings or to surrounding soil, the more contrasting substrates used in this work. Plant growth parameters (Fig. 4A), and content of different pigments (Fig. 4B) were measured 18 weeks after transplantation. All growth parameters, excepting root dry weight, displayed significantly lower levels when plantlets were transplanted to fresh Tambillos tailings compared to surrounding soil.

**Figure 5:**
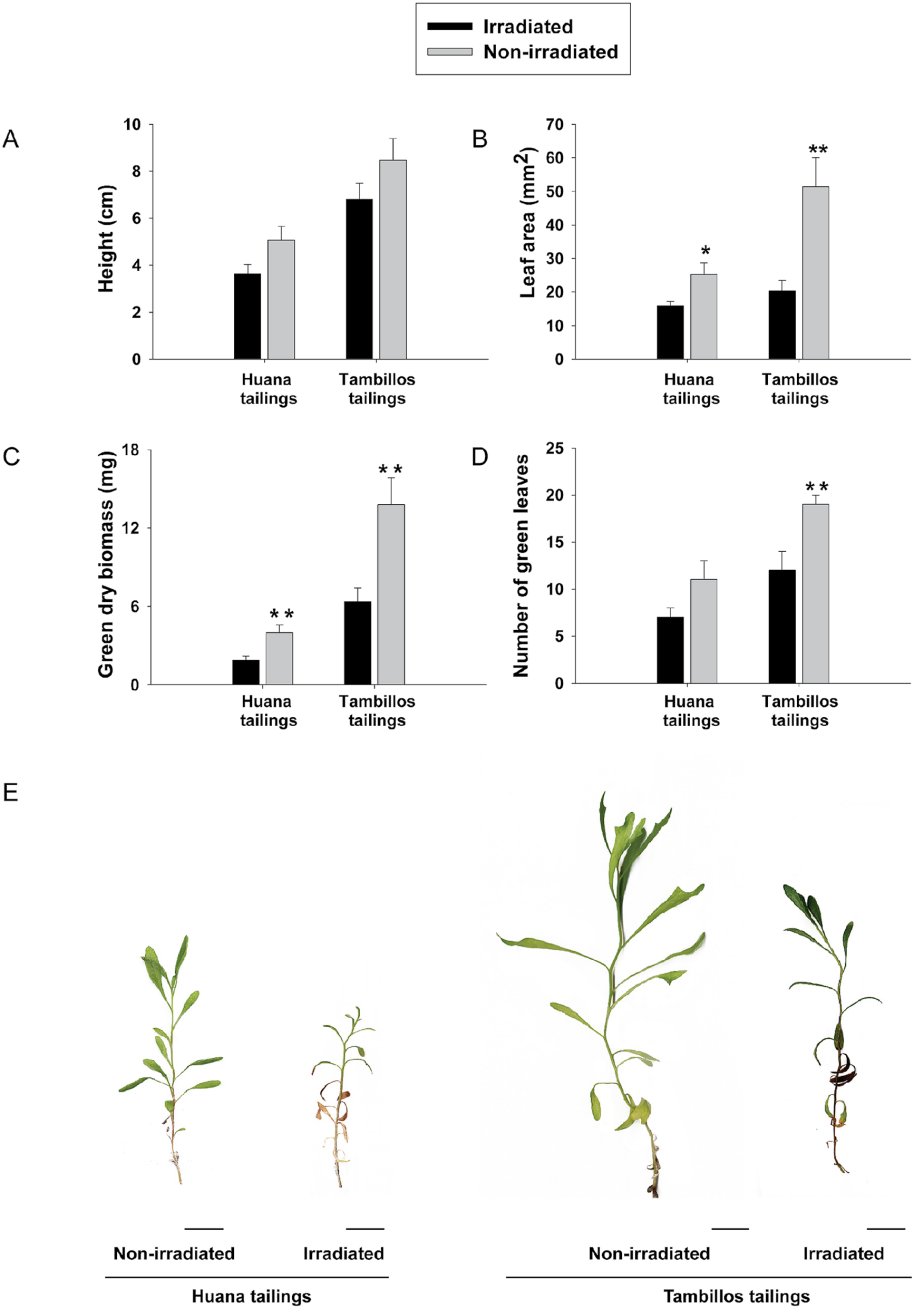
Effects of the microbial community in the establishment of *Baccharis linearis* plants in non-irradiated or irradiated tailing substrates. Plantlets were transferred to non-irradiated (grey bars) or irradiated (black bars) tailing substrates six weeks after sowing and plant growth parameters **(A)** height; **(B)** leaf area; **(C)** green dry biomass and **(D)** number of green leaves, were measured 18 weeks after transplantation. Bars represent average ± SE (24 replicates per treatment). Asterisks indicate significant differences according to a one-way ANOVA (for parametric data) or to a Kruskal–Wallis test (for non-parametric data) considering irradiation as factor; *p < 0.05 **p < 0.01. **(E)**. Representative plants grown on each treatment are shown, bars indicate 1 cm.

In a second approach, the effects of substrate irradiation (heavily diminishing soil microbial community) were also measured in seedlings establishment. In this case, six weeks after germination seedlings were transferred to non-irradiated and irradiated substrates, and growth parameters were measured 18 weeks later (the longest time tested). No differences were observed in plant heights regarding irradiation treatment (Fig. 5A,E) but plants had leaves significantly bigger when grown in a non-irradiated substrate (Fig. 5B,E). As a proxy of plant health, only the green leaves were weighted (Fig. 5C), counted (Fig. 5D) and compared in both treatments, finding a tendency to lower values when plants were grown in irradiated substrates.

As a final approach, germination and growth were measured when seeds were germinated from the beginning in irradiated or non-irradiated tailing substrates. Here, germination rates and growth parameters were measured after seven weeks. Germination rates ranged between 67 and 76% and non-significant differences were observed if substrates were previously irradiated (Table 3). However, plant height, number of leaves, secondary root numbers and dry biomass (shoot and roots) were significantly higher when seeds were sown in non-irradiated substrates (Table 3). When results from both non-irradiated sites were compared, differences were observed only in few parameters: shoot dry weight was 1.3 times higher in Tambillos tailings (T = − 3.21; p < 0.01) and primary roots were 1.4 times longer in Huana tailings (T = 4.52; p < 0.01). Plants grown in both irradiated substrates showed differences in root dry weight (almost 2.0 times higher in Tambillos tailings; T = − 4.45; p < 0.01); number of secondary roots (2.2 times higher in Huana tailings; W = 172; p = 0.03) and in root/shoot ratio (2.2 times in Huana tailings; W = 99; p < 0.01) (Table 3).

**Table 3:** Germination rate and plant-growth parameters in *Baccharis linearis* plants sown and grown in non-irradiated or irradiated tailing substrates from Huana and Tambillos.

Study sites	Growth parameters^**†,**^^‡^	Non-irradiated substrate	Irradiated substrate	Statistic	*p*-value^¶^
*Huana*	Germination (%)	71.6 ± 4.5	67.6 ± 6.6	T = − 0.34	0.7356
	Height (mm)	22.9 ± 0.8	15.0 ± 0.7	T = − 7.38	**
	Number of leaves	7.3 ± 0.1	5.0 ± 0.2	W = 1294.5	**
	Primary root length (mm)	36.8 ± 1.3	28.3 ± 2.2	T = − 3.46	**
	Number of secondary roots	1.9 ± 0.3	1.2 ± 0.2	W = 253.5	*
	Shoot dry weight (μg)	382.0 ± 19.6	195.1 ± 30.8	T = − 5.11	*
	Root dry weight (μg)	69.8 ± 6.4	32.1 ± 3.0	T = − 5.77	**
	Root/shoot ratio	0.2 ± 0.0	0.2 ± 0.0	T = 0.25	= 0.8088
*Tambillos*	Germination (%)	76 ± 4.5	67.2 ± 4.5	T = − 1.41	= 0.1757
	Height (mm)	23.6 ± 0.9	14.7 ± 0.6	T = − 8.37	**
	Number of leaves	7.8 ± 0.2	5.3 ± 0.2	W = 1314.5	**
	Primary root length (mm)	27.2 ± 1.8	28.8 ± 3.7	T = 0.13	= 0.8973
	Number of secondary roots	2.7 ± 0.4	0.6 ± 0.2	W = 130	**
	Shoot dry weight (μg)	496.4 ± 29.9	172.9 ± 18.5	T = − 9.22	*
	Root dry weight (μg)	92.5 ± 9.9	61.5 ± 6.1	T = − 2.62	*
	Root/shoot ratio	0.2 ± 0.0	0.4 ± 0.0	T = 5	*

^**†**^Plant-growth parameters were measured seven weeks after sowing.

^‡^Values represent averages ± SE of 50 replicates per treatment.

^¶^Significant differences considering irradiation as factor, T-Student test (T) or Wilcoxon (W).

*p < 0.05.

**p < 0.01.

After 18 weeks (second approach), four seedlings grown on each substrate were randomly selected to detect culturable endophytic and rhizospheric microorganisms. Endophytic microorganisms were not detected in any analyzed tissue. Similar levels of colony forming units (cfu/g^-1^ of roots) were detected in the rhizosphere of irradiated and non-irradiated Huana tailing substrates [log 8.73 (± log 0.46), and log 8.96 (± log 0.11), respectively]. Plants grown in irradiated.

Tambillos tailings had significantly higher colony forming units than those grown in the non-irradiated conditions: [log 9.91 (± log 0.43) vs. log 7.86 (± log 0.23), respectively]. Phenotypically, rhizospheric colonies were more diverse in the non-irradiated substrates (4–6 different colonies) than in the irradiated substrates (2–3 different colonies). On the other hand, the presence of arbuscular mycorrhizae was not detected in the roots grown in irradiated substrates (Supplementary Fig. S2A and B online, respectively). However, arbuscular mycorrhizae were detected in roots of seedlings grown in non-irradiated substrates (Supplementary Fig. S2C-F online). Comparing results in non-irradiated substrates, arbuscular mycorrhizae abundance and colonization were significantly higher in Huana tailings (69.42% ± 4.25 and 85.45 ± 5.44, respectively) than in Tambillos tailings (33.81% ± 4.32 and 50.47% ± 7.75, respectively). Arbuscular mycorrhizae were isolated from both tailings, and surrounding soil by trapping methods using *Tagetes *sp*.* and *Hordeum vulgare* as hosts (Supplementary Material online), obtaining seven arbuscular mycorrhizae spore isolates. Taxonomic affiliation revealed that all isolates belonged to *Glomus* genus, being *Glomus intraradices* the closest species matching in the databases (99% identity).

## Discussion

Some of the challenges that plants face in colonizing tailings were analyzed in two copper mining tailings from operations located in northern-central Chile, using the pioneer plant *B. linearis*. The role that *B. linearis* associated microorganisms play in plant germination and establishment was especially assessed. We found that native microbiota was required to improve the pioneer plant *B. linearis* establishment and growth in copper tailings, and that microbial communities were more influenced by the pioneer plant’s presence than the substrate physicochemical properties.

In contrast with reported acid/alkaline conditions in copper tailings, physicochemical analyses of tailings used in this work demonstrated that very little or no secondary acidification had occurred. This may be explained, at least in part, by high evapotranspiration rates occurring at the upper sulfide-rich horizon of tailings, due to arid and semiarid climate conditions, and/or high content of dissolved CO_3_^2-^ due to high calcite/pyrite ratio, which is typical in these tailings [3,]. Since pyrite is the main source of sulfide in porphyry copper deposits, the latter does not only imply a high buffering capacity of the substrate solution, but also a lower susceptibility to iron sulfide oxidation and to the subsequent production of acid mine drainage^21^. On the other hand, the presence of organic residues coming from flotation reagents can explain the high organic matter content in the fresh tailings^22^. High soluble sulfate levels found in all these tailings could derive from the high amount of solubilized sulfate-containing minerals, like gypsum and jarosite. These minerals are commonly found in the Chilean copper mining area^23^, and not necessarily are related to sulfide oxidation, as for acidic tailings^24^. These high amounts of sulfate can also explain elevated concentrations of exchangeable cations, due to the formation of stable complexes with SO_4_^2-^, thus preventing precipitation after interaction with other anions^21^. Analysis of cation concentrations in rhizosphere substrates showed that both tailings had sodium ion levels equivalent to those of sodic soils^25^. This is relevant as such ions are transported to the surface of the tailings and, as water evaporates, precipitated as sodic salts. This salinization is evidenced by high electrical conductivity levels, and in fact, high levels were found in the three tailings when compared to surrounding soil, especially in fresh Tambillos tailings (Table 2). Electrical conductivity values of these tailings are within the range that usually limits the growth of terrestrial plants^18^. Comparatively low cation exchange capacity and total organic carbon values were obtained for both tailings when compared to surrounding soil. These parameters are slightly more favorable towards plant colonization in the older Tambillos substrate. This is consistent with longer time of weathering, surface deposition of soil particles from surrounding fields, and higher vegetation coverage. Differences between tailings and surrounding soils have also been reported for copper, zinc, and iron levels in Chilean copper mining tailings^26^. However, no differences could be detected among tailing locations, and total metal values were in the range of two (iron and zinc) to five (copper) times higher than those of surrounding soil.

Microbial community compositions were studied to better understand *B. linearis* establishment in copper mining tailings. For logistic considerations, the selected culture-independent technique was chosen to make prospective comparisons of microbial communities in these soil substrates. It should be kept in mind that this molecular technique is not as powerful in coverage (richness) as high-throughput, next-generation sequencing approaches, since only dominant (abundant) members of the respective microbial community are detected. Therefore, future studies are required to get a deeper understanding of the *B. linearis* microbial community dynamics and composition in these substrates. The comparison of the most abundant members composition of the bacterial and fungal community suggested that substrate conditions have a strong influence in these communities (Fig. 3). This was mainly observed in fresh Tambillos tailings, which clearly differentiates from all other substrates, especially in terms of beta diversity. Huana and Tambillos tailings showed lesser but evident changes according to aging and/or compartments (Fig. 3). The clear differentiation of beta diversity in Tambillos tailings relative to Huana tailing and surrounding soil contrasts with the trend of physicochemical differences discussed above. Tambillos tailings were more like surrounding soil than to Huana tailings (Table 2, Supplementary Table S1 online). This suggests that these microbial communities are less affected by differences in electrical conductivity, cation exchange capacity, and total organic carbon. The latter may be also true for metal concentrations in both tailings, as these microbial communities were previously reported not to be substantially variable among tailing locations, but significantly different in surrounding soil^26^. This would imply that metals have a low influence in the microbial diversity among tailings, but still affect plant growth^27^, although there are reports of contrasting evidence^28^. On the other hand, the microbial community similarity between Huana tailings and surrounding soil can also be explained by significant and continuous microbial inoculation from surrounding soils^29^, probably by soil erosive processes^30^.

This is the first report approaching beta diversity analyses of microbial communities from abandoned copper mining tailings. In addition to the unexpected results just discussed, this analysis was also able to shed some light on what is driving beta diversity of both tailings, and surrounding soil *B. linearis* rhizoplane microbial communities. Differences in total beta diversity index (β_SOR_) were found for Tambillos tailings with respect to Huana and surrounding soil (Fig. 3). Turnover patterns (β_SIM_), as well as β_SOR,_ mainly distinguished Tambillos from Huana tailings and surrounding soil (Fig. 3B). This indicated that species replacement (turnover) was more significant than non-replacement (nestedness) in Tambillos tailings. However, high species richness was found for these tailings, which is in agreement with literature, indicating that bacterial and fungal diversity indices tend to be higher in old, reclaimed sites (15 to 20 –years old), as recovery time increased^31^. Therefore, higher turnover values for Tambillos tailings could be explained by a combination of factors as compared to Huana tailings, including a larger size of particle material (texture %). These factors increase water infiltration, together with high dissolved organic carbon, and low cation exchange conductivity, and electric conductivity values. Therefore, it may result in an improvement of micro-environmental conditions perceived by microbial communities.

In any case, it is clear that different physicochemical conditions among substrates do not necessarily explain changes in *B. linearis* rhizosphere and non-rhizosphere microbial communities. This is further demonstrated when gross changes in microbial community structures are analyzed. While bacterial community structures were mainly affected by “root compartment” (microenvironment conditions), fungal communities were clearly influenced by “location” (geographical conditions) (Fig. 1). In addition, only rhizoplane bacterial community structures were significantly different from the rhizosphere, or non-rhizosphere environment. The latter suggests that close association with root plants provides a microenvironment different enough to influence *B. linearis* associated bacteria. Processes mainly operating at a few microns range (distinguishing rhizoplane from rhizosphere microhabitats), especially under conditions when fluid transport is limiting, may explain the preceding observation. Vegetation development causes the incorporation of organic carbon (rhizodeposition), stimulating microbial activity and element cycling processes^32^. In turn, root exudates production by pioneer plants may generate differential activation of rhizobacteria^33^. Thus, *B. linearis* may play a selective role at their rhizoplane, shaping bacterial diversity. This is in agreement with the proposed two-step selection model for root microbiota differentiation where plant ecotypes first restrict access to rhizoplane to certain specific microbiota members, and then the endophytic association may take place^29^. In contrast, fungal communities may overcome short-range rhizospheric microhabitat limitations by extensive filamentous growth, and therefore being relatively more affected by long-range space (location) and time (aging) conditions.

Which bacterial taxa are differentially associated with *B. linearis* colonizing copper mining tailings? Previous reports had shown changes in relative abundances of *Acidobacteria*, *Firmicutes, Nitrospira, Alphaproteobacteria, Gammaproteobacteria,* and *Deltaproteobacteria*^5,9,10,34,35^. In partial agreement with these reports, this work revealed that *Proteobacteria*, *Actinobacteria*, *Firmicutes*, and *Bacteroidetes* taxa were found abundant in all tailings, although the composition of predominant phyla differed across the sites (Fig. 2 and Supplementary Fig. S1 online). In addition, this work further demonstrated that the rhizoplane and rhizosphere of *B. linearis* were clearly dominated by *Proteobacteria, Firmicutes*, and *Actinobacteria* in both tailings and in the surrounding soil. In rhizoplane and rhizosphere compartments, plants locally provide higher levels of C and N sources than levels that can be found in tailings, potentially favoring *Proteobacteria*, which can play a wide range of roles in the C, N and P cycles^31^. *Gammaproteobacteria* was a ubiquitous and abundant class in root compartments of *B. linearis* across all locations. *Betaproteobacteria* showed a marked presence at rhizoplane microhabitats, while *Alphaproteobacteria* were relatively less abundant, irrespective of the rhizosphere or non-rhizosphere microhabitats. Detection of members of the *Firmicutes* phylum in these substrates can be explained due to their tolerance to very low nutrient levels. Detection of *Actinobacteria* phylum was expected since it has long been recognized that several members within this phylogenetic group are capable to carry out biological nitrogen fixation among other microbially driven soil processes^34^.

This work also reports a poor performance in plant growth and establishment when *B. linearis* individuals were grown in fresh Tambillos tailings, as compared with those grown on surrounding soil (Fig. 4). Besides obvious physicochemical differences between these substrates, plant growth disparities may also be explained by observed differences in bacterial and fungal community structures (Fig. 1). The same might apply to differences in plant growth between fresh and older tailings of the same mine (fresh vs. old Tambillos tailings), suggesting that ongoing processes of primary ecological succession are taking place^35^. Small particle sizes found in these tailings (Table 2) contribute to higher bulk density, mechanical compaction, and smaller pore size, hampering water infiltration, and therefore, plant growth.

The role of associated microbiota on *B. linearis* growth and establishment was further explored in irradiated tailings with significantly depleted microbial activity. The irradiation method is recommended over the use of antibiotics or autoclaving, because it effectively reduces microbial community while producing low changes in the physicochemical properties of substrates^36^. Three consecutive doses of 25 kGy were used as they have been reported to reduce fungal, bacterial, and algal abundances in soils^36,37^. It has to be noted that irradiation treatment does not permanently eliminate microorganisms. The re-colonization process in sterilized/irradiated substrates has been studied, reporting an increase in microorganisms’ abundance over time, although with decreased diversity^37,38^. Although 18 weeks after irradiation total colony forming unit values for irradiated substrates were in fact higher than in non-irradiated ones, diversity of these colonies was significantly lower, and therefore, not affecting the outcomes of this work.

In any case, irradiation of tailings had a severe effect on plant growth (Fig. 5), which can be explained by the absence (although transitory) of an active and diverse, normally associated, microbiota. In a similar way, plant growth on fresh Tambillos tailings (Fig. 4) was clearly affected by the presence of a quite different microbial community compared with those of abandoned tailings or surrounding soil (Fig. 3). As irradiated substrates contained significantly lower available N levels (Supplementary Table S4 online), this limitation can contribute directly to impaired plant growth, more than the sole absence of normal microbiota. The ability of plants, including *B. linearis*, to manage N limitation under conditions present in tailings is probably quite restricted, especially without the activity of N-fixing members of rhizosphere microbiota. Relatively high numbers of *Alphaproteobacteria* and *Betaproteobacteria* found in *B. linearis* rhizosphere individuals colonizing these tailings is consistent with a need for N-fixing proteobacterial classes^39^.

An additional restriction for growth on irradiated substrates might come from P access, not because of decreased availability, as P levels were not reduced by irradiation (Supplementary Table S4 online). The absence of arbuscular mycorrhizae, otherwise abundant in *B. linearis* plants colonizing non-irradiated tailings (Supplementary Fig. S2 online) may explain this growth effect. Fungal spore isolation from tailings colonizing plants and further characterization clearly showed a massive presence of arbuscular mycorrhizae belonging to the *Glomus* genus. This is recognized as a phosphate solubilizing fungal group^40^, able to produce changes in rhizospheric microbial communities^41^. It is worth mentioning that interaction between mycorrhizal fungi and associated bacteria has been demonstrated to protect plant species from heavy metals effects^42^, besides helping in nutrient acquisition^43^, and phytostabilization of copper tailings^44^.

## Methods

### Site description

Plants, substrates, and soils were obtained from two copper mines: Tambillos (30° 12′ 06" S, 71° 14′ 27" W) and Huana (30° 42′ 24" S, 70° 57′ 20" W), near the cities of Coquimbo and Ovalle, respectively, north-central Chile (Table 1, Supplementary Fig. S3 online). Coquimbo has a coastal steppe climate whereas Ovalle has a desert one. Cu tailings in this region came from mines that processed porphyry Cu, using alkaline foam flotation processes^3^. Except for the surrounding soil used as a control (an Aridisol), all the soils collected were Technosols, because they had been differentially impacted by anthropogenic activities. Because of their high levels of sulphidic material, these soils can be also classified as Thionic-Technosols^45^.

### Soil, substrate, and plant sampling

Three copper mining tailings were selected (Table 1). The first two were fresh Tambillos and old Tambillos obtained from an operation closed and abandoned in 1983, respectively, both containing sulphidic material from a copper/gold flotation procedure^26^. The third site came from Huana tailings abandoned in 1998, containing sulphidic material from a copper flotation procedure^7^. A composite of four randomly selected points was taken from each dump. Except in fresh Tambillos tailings, where no plant colonization had already occurred, a significant level (30–50% of total plants) of *B. linearis* individuals had been reported in these tailings^46^. The surrounding soil sample, selected as a control, also a composite of four randomly selected points where colonizing *B. linearis* individuals were found, was obtained next to the Huana site (Table 1). *B. linearis* is a common native species in north-central Chile with no conservation problems. There are no national regulations for collection of plant material, but seed collection followed scientific national proposed guidelines for wild species^47^.

For rhizoplane microhabitat studies, roots of *B. linearis* were collected in separate sterile propylene tubes. One and a half kg of rhizosphere from *B. linearis* and non-rhizosphere substrates were also collected in sterilized bags. Rhizoplane and rhizosphere were differentiated from non-rhizosphere (not in contact with plants) material, as the former corresponds to material firmly attached to root surface whereas the latter is only loosely attached (remaining associated to roots after gentle shaking). The non-rhizosphere substrate was obtained from areas without vegetation, at a distance of 1.5 m from sampled plants. Rhizosphere and non-rhizosphere samples were taken at 0–20 cm depth, using a stainless-steel shovel. Four root samples per plant were taken, and substrate considered as rhizosphere was obtained from a distance of 10 cm, as a maximum. Fresh Tambillos substrates were considered as non-rhizosphere samples. All samples were immediately transported to the laboratory, sieved (mesh size 5 mm), and stored at 4 °C. Culture dependent and physicochemical characterizations were performed in the first 2–3 days. Stored substrates were used for plant growth tests no later than two months, at most.

### Physico-chemical analysis of tailings and soil substrates

Sample preparation was carried out according to standard procedures^48^. All samples were dried in an airflow chamber at 30ºC until constant weight. Dry samples were passed through a 2 mm pore size sieve prior to analyses. pH values in water:soil (1:1) extracts were measured according to USDA^48^, and water soluble organic carbon was determined by the wet combustion method^49^. A saturated water extract (2.5:1 water:soil) of each sample was prepared for the determination of water soluble organic carbon, electrical conductivity and sulfate concentration. Sulfate was determined by turbidimetric analysis of the amount of BaSO_4_^50^. Cation exchange capacity was evaluated by saturating (1:2.5 substrate:extractant solution) samples with ammonium acetate (1 N). Texture was determined using the hydrometer method described by Gee and Bauder^51^.

### Plant growth parameters analysis

Height, the number of leaves, primary root length, and the number of secondary roots were determined using the largest plant in each tube/pot. Height and primary root lengths were determined by scanning plants using the ImageJ software (NIH, USA^52^). Dry weight was estimated using all available plants in each tube/pot, drying seedlings at 40 ºC to constant weight, and using an analytical scale. Foliar areas were measured selecting three leaves from ten plants per condition. Leaves were scanned and images were processed with Adobe Photoshop CS version 8.0.1. Photosynthetic pigment contents were determined using adult leaves (between the third and fifth leaf from the apex). Three to four mg of fresh leaves were weighed in an analytical scale and treated with dimethylformamide for 24 h, at 4 ºC in the dark. After that, absorbance was measured at 664, 647, and 480 nm with a GeneQuant 1300 spectrophotometer. Chlorophyll a, b, and carotenoids contents were calculated according to Wellburn^53^.

### Tests of microbial effects on the germination and growth of *B. linearis* in copper mine tailings

*B. linearis* seeds, obtained from plants collected in Tambillos tailings, were surface sterilized for 1 min in ethanol 70%, washed three times with sterile water, treated for 1 min with H_2_O_2_ (4.5%), and washed five times with sterile water. Seeds were then stratified in sterile water during 48 h in the dark at 4 ºC. Stratification allows better seeds germination, mimicking natural conditions. To evaluate growth and establishment of *B. linearis* seedlings, sterilized seeds were sown in Magenta flasks with sterile perlite in diluted Hoagland solution and grown in a controlled lab environment plant growth chamber (23 ± 2 ºC; 12/12 h light/dark; light intensity of 103 ± 14.3 μm s^−1^ m^−2^). After three weeks, seedlings were transferred to pots (five per pot) containing 100 g of substrate.

To assess the role of microbial community in the establishment of *B. linearis* plants, seedlings were transferred to pots as indicated above, but this time containing 100 g of non-irradiated or irradiated tailings. Irradiation was accomplished with three doses of 25-kGy gamma irradiation, using Co^60^^54^. Irradiation did not produce significant changes in nutrients other than a decrease in organic matter and available nitrogen (Supplementary Table S4 online), as gamma irradiation destroys organic compounds, including nitrogenated compounds. As expected, irradiation produced an almost complete loss of viable cell counts (less than 10^2^ colony forming units g^-1^ of irradiated material). In all growth of plantlets tests, substrates were irrigated at 50% field capacity according to ISO^55^. Each treatment had 24 replicates. This number of replicates is based on similar studies to adequately address experimental variability in plant experiments, to improve statistical support^15,56^, and were processed and measured in three 8-replicates blocks. For tests of seed germination, 15 g of each substrate (irradiated or not) were transferred to 50 mL sterile tubes and watered with sterile water at 50% field capacity. Five seeds were sown in each growth substrate as described above, and plant growth parameters were measured after seven weeks. The chosen time allowed determination not only of germination rates but also other plant development features. Each treatment (non-irradiated and irradiated) had 24 replicates.

### Viable microbial counts analysis

Four plants from each treatment were randomly selected to detect endophytic or rhizospheric microorganisms in leaves (2–3 per plant), shoots (two segments of 2 cm per plant), and roots (four segments of 2 cm per plant). Roots were submerged in sterile 1 mM MgSO_4_ and sonicated to extract the substrate and microorganisms from the rhizoplane. Then, all tissues were weighed and sterilized with 2% NaOCl for 2 min under sonication, and 8 min without sonication. After that, samples were washed three times with sterile water. The third wash water was kept to confirm sterility and discard viable counts arising as endophytic false positives from non-endophytic cells that remained attached to the tissue after the previous treatment. Tissues were ground in sterile mortars using 0.5 mL of 1 mM MgSO_4_, and 2% w/v polyvinylpyrrolidone. Samples were plated in serial dilutions with two replicates in R2A or 869 media and placed at 30 ºC during seven days prior to colony counting.

### DNA extraction, PCR amplification, and T-RFLP analysis

Metagenomic DNA was extracted as previously described^37^, from 0.5 g of all substrate samples and root compartments (rhizoplane, rhizosphere, and non-rhizosphere). Four different DNA extractions were performed and analyzed for each sample. Metagenomic DNA was used as a template for bacterial 16S rRNA genes PCR amplification using oligonucleotide primer pair 27F-B (5′-AGRGTTYGATYMTGGCTCAG-3′)^57^ labeled with fluorochrome 6-FAM at 5′ end, and 1392R (5′-ACGGGCGGTGTGTRC-3′)^58^. For fungal microbial community analysis, metagenomic DNA was used as a template for PCR amplification of internally transcribed spacer region using oligonucleotide primer pair ITS1F (5′-CTTGGTCATTTAGAGGAAGTAA-3′)^59^ labeled with fluorochrome HEX at 5′ end, and ITS4 (5′-TCCTCCGCTTATTGATATGC-3′)^60^. Each PCR mix reaction was performed in a final volume of 25 μL (in 10X PCR buffer). For bacterial 16S rRNA genes amplification, the PCR mix and PCR conditions have been previously described^37^. For internally transcribed spacer region amplification, the mix contained 0.3 µM of each primer, 0.25 mM of each deoxynucleotide triphosphate, 2 mM MgCl_2_, 0.4 μg μL^−1^ of bovine serum albumin, and Taq polymerase (0.25 U). For the internally transcribed spacer region, PCR conditions were 94 °C for 5 min, 13 cycles of 95 °C for 35 s, 55 °C of annealing temperature for 55 s and 72 °C for 45 s, followed by 13 and 9 cycles of 2 and 3 min of increased elongation time, respectively, and a final extension step of 72 °C for 7 min. To avoid DNA contamination, PCR mixtures were irradiated with UV light for 5 min before the addition of Taq polymerase. As most T-RFLP profiles showed the same trends, only results from *Hha*I profiles are reported.

### T-RFLP data handling and putative assignation of terminal restriction fragments analysis

Terminal restriction fragment sizes raw data were handled as described before^37^. The composition of each microbial community was described as operational taxonomic units, represented by terminal restriction fragments. As only relative abundances for each operational taxonomic unit were available, these data qualify as compositional^61,62^. Assignation of terminal restriction fragments to bacterial taxa was performed using the Ribosomal Database Project (http://rdp8.cme.msu.edu/html), and the National Centre of Biotechnology Information database (http://trflp.limnology.wisc.edu/index.jsp). Results from both databases were essentially the same. Only terminal restriction fragments unequivocally assigned to specific bacterial taxa were considered. This explains the high percentage of unmatched terminal restriction fragments.

### Statistics

Statistical analyses for plant growth parameters were carried out with InfoStat software (Córdoba, Argentina). A variance analysis using the absolute value of residues as the dependent variable was performed to test normality and homogeneity of variances, using Shapiro-Wilks and Levene tests, respectively. When required, data were transformed using log_10_, square root, or arccosine. T-Student test (T) or ANOVA was used for parametric data. An LSD Fisher post-hoc test followed ANOVA tests (α = 0.05). For non-parametric data, Wilcoxon (W) or Kruskal–Wallis (K) tests were utilized with 95% significance.

To determine taxonomic composition of microbial communities, as well as similitudes and changes among spatial distribution, several statistical analyses were performed. R packages were used for statistical analysis of Alpha and Beta diversity and Beta diversity partition^37^. Beta diversity (β_SOR_), the number of changes in species composition along an environmental gradient^63^, was assessed considering its two components: species replacement or turnover (β_SIM_), and species loss or nestedness (β_SNE_), as previously detailed^37^. Discretized (presence/absence) T-RFLP profiles from both 16S rRNA gene and internal transcribed spacer sequences were coupled to generate a single microbial community dataset (meta-community analysis). NMDS graphs were obtained based on Sørensen (β_SOR_) dissimilarity indices. Multiple-site dissimilarity was determined computing β_SOR_, β_SNE_ and β_SIM_ indices, and NMDS analyses were used to visualize grouping or segregation of samples. Density plots were performed to evaluate whether turnover or nestedness is the predominant process in microbial composition^64^. Beta diversity of bacterial and fungal communities (16S rRNA and internal transcribed spacer terminal restriction fragments, respectively) was independently analyzed. To do so, the multivariate statistical software Primer v6 (Primer‐E, Plymouth, UK) applying Bray–Curtis distance matrices based on the square root-transformed abundance of each terminal restriction fragment was used. NMDS analyses grouping data according to their similarity and two-way ANOSIM were performed, examining statistical significance of grouping by factors defined as localization and root compartment^20^.

## Supplementary Information

Supplementary Information.
