# Supplementary material for: The microbial community from the early-plant colonizer (Baccharis linearis) is required for plant establishment on copper mine tailings
Source: Sci Rep. 2021 May 17;11:10448. doi: 10.1038/s41598-021-89769-1 (PMC8129112; doi:10.1038/s41598-021-89769-1)
Supplement: Supplementary file 1 — Supplementary Information. [file 41598_2021_89769_MOESM1_ESM.docx]

**SUPPLEMENTARY MATERIAL**

The microbial community from the early-plant colonizer (*Baccharis linearis*) is required for plant establishment on copper mine tailings

Consuelo Gazitúa, Verónica Morgante, María Josefina Poupin, Thomas Ledger, Gustavo Rodriguez-Valdecantos, Catalina Herrera, María del Carmen A. González-Chávez, Rosanna Ginocchio, Bernardo González

**Fig. S1: More abundant bacterial taxa from the rhizoplane of *Baccharis linearis* based on 16S rRNA clone libraries sequences.** The relative abundance of each taxonomic group is an average (indicated in parenthesis) of all locations (Huana, Tambillos and surrounding soil).

*Cloning, sequencing and sequence analysis of 16S rRNA genes*

Three clone libraries were constructed using the 16S rRNA gene PCR products of the rhizoplane compartment from *B. linearis* plants growing in HT, TT and surrounding soil. Cloning procedures were performed as previously reported (Morgante *et al*., 2010). Briefly, PCR products were mixed and purified with Qiaquick PCR purification kit (Qiagen, Hilden, Germany). Cloning procedures were performed using TOPO TA cloning kit (Invitrogen, MA, USA) and the chemically competent *E. coli* cell One Shot Mach1™-T1R (ThermoFisher Scientific, MA, USA) according to manufacturer’s recommendations. White colonies growing in Luria Bertani agar supplemented with 50 μg mL^-1^ kanamycin were selected. The plasmids of positive clones were directly extracted from a colony and amplified using the primer pair pUC/M13F and pUC/M13R provided by the kit. Positive PCR products were purified (Qiaquick PCR purification kit, Qiagen, Hilden, Germany) and sequenced using the primer pair 800R (5'-TACCAGGGTATCTAATCC-3') and 518F (5-CCAGCAGCCGCG GTA AT-3’) and the automated DNA sequencer*,* ABI3730XL (Macrogen Inc*.,* Seoul, Korea)*.* The similarity of these sequences with the closest relatives described in the NCBI database was in the range 80-99%; 80-90% for most of the cultivated species, and 97-99% for most of uncultivated species.

One hundred and twenty-four 16S rRNA gene clones (45 originated from HT; 48 from TT and 31 from surrounding soil) were partially sequenced (500-800 bp). Bacterial diversity coverage achieved by clone sequencing was evaluated with rarefaction analyses assuming that one operational taxonomical unit is formed by the sequences that present a similarity equal to or greater than 97% (Zaballos *et al.,* 2006). This analysis indicated higher than expected bacterial sequence diversity, as no asymptotic curves were observed. Although an additional sequencing effort would be required to obtain a better coverage on the diversity of the amplified 16S rRNA genes, it was assumed that at least the most abundant phylotypes thriving in *B. linearis* RP could be identified.

Morgante, V., López-López, A., Flores, C., González, M., González, B., Vásquez, M., Rosselló-Mora, R., Seeger, M. (2010). Bioaugmentation with Pseudomonas sp. strain MHP41 promotes simazine attenuation and bacterial community changes in agricultural soils. FEMS Microbiol. Ecol. 71, 114–126. *Erratum:* FEMS Microbiol. Ecol. (2010). 72, 152.

Zaballos, M., López-López, A., Ovreas, L., Bartual, S.G., D'Auria, G., Alba, J.C., Legault, B., Pushker, R., Daae, F.L., Rodríguez-Valera, F. (2006). Comparison of prokaryotic diversity at offshore oceanic locations reveals a different microbiota in the Mediterranean Sea. FEMS Microbiol. Ecol. 56, 389–405.

**Fig. S2: Detection of arbuscular mycorrhizal (AM) fungi in *Baccharis linearis* roots grown in non-irradiated or irradiated tailings-derived substrates.** Roots were stained with trypan blue to detect AM). Photographs of representative roots from Huana (A, C and E) or Tambillos tailings dump (B, D and F) after grown on irradiated substrates (A and B) or in non-irradiated substrates (C-F). Bars correspond to 0.1 mm (A-D) and 0.02 mm (E and F).

*Arbuscular mycorrhiza detection and quantification*

Roots from four plant individuals per treatment were clarified with KOH 10% w/v at room temperature during two days. If necessary, they were washed with sterile water and treated with H_2_O_2_ 6% w/v (1 to 3 min). Cleared roots were then treated with HCl 10% w/v during 3 min. After that, HCl was removed and roots were stained with Trypan blue 0.05% in glycerol 50% v/v, during two days at room temperature and finally the stained roots were transferred to glycerol 50% v/v. Roots were cut every 1 cm and visualized under a microscope (25 sections per root). Arbuscule abundance and the colonization intensity were determined following the methodology described by Trouvelot et al. (1986) and data were analyzed using the Mycocalc program (<https://www2.dijon.inra.fr/mychintec/Mycocalc-prg/download.html>). Arbuscular mycorrhiza spores from the different substrates were also extracted using the modified wet sieving method by filtering the spores stratified with the mesh sizes of 300, 100 and 45 μm. The isolation of fungal spores was performed by a trapping method as reported (Brundrett *et al.,* 1994). Briefly, 200 g of sterile sand were poured into 300 mL pots, followed by a layer of 1.5 g of rhizospheric substrate samples (HT and TT and surrounding soil) and covered by 100 g of sterile sand. *Tagetes* sp., an annual herbaceous plant from the *Asteraceae* family, was selected as a trap plant due to its ability to colonize mine tailings (Carrillo-González & González-Chávez, 2006) in association with mycorrhizal fungi such as *Glomus* sp. and *Scutellospora* (González-Chávez *et al.,* 2009; Ortega-Larrocea *et al.,* 2010). Pots with one to three plants were placed in a greenhouse and watered three times a week with modified Hoagland solution. After 16 weeks, AM spores were collected by a wet sieving method and observed under stereoscopic microscope, as described above. Finally, individual, pure cultures of the previously obtained AM were carried out in 1 L pots filled with sterile sand, using *Hordeum vulgare* as a host plant. This grass, from the *Poaceae* family, was selected based on its adaptability to greenhouse conditions, its extensive and uniform root system and high mycorrhizal dependency (Chaurasia & Khare, 2006). Pots were kept at the same environmental conditions described for the other assays, and watered three times a week with modified Hoagland solution. After 6-month of incubation, 50-100 spores were collected in aseptic conditions from each treatment and used to extract DNA using the FastDNA spin kit for soil (MP Biomedicals, Santa Ana, California, USA), as described above. For genetic preliminary taxonomical identification, partial 18S rRNA genes were amplified by nested PCR as previously reported (Lee *et al.,* 2006).

Brundrett, M., Melville, L., Peterson, L. (1994). Extraction and staining of hyphae from soil. Practical Methods in Mycorrhiza Research. Mycologue Publications, Waterloo, 71-80.

Carrillo-González, R., González-Chávez, M.C.A. (2006). Metal accumulation in wild plants surrounding mining wastes. Environ. Pollut. 144, 84–92.

Chaurasia, B., Khare, P.K. (2006). *Hordeum vulgare*: a suitable host for mass production of arbuscular mycorrhizal fungi from natural soil. Appl. Ecol. Environ. Res. 4, 45–53.

González-Chávez, M.C., Carrillo-González, R., Gutiérrez-Castorena, M.C. (2009). Natural attenuation in a slag heap contaminated with cadmium: the role of plants and arbuscular mycorrhizal fungi. J. Hazard. Mat. 161, 1288–1298.

Lee, J., Park, S.H., Eom, A.H. (2006). Molecular identification of arbuscular mycorrhizal fungal spores collected in Korea. Mycobiol. 34, 7-13.

Ortega-Larrocea, M.P., Xoconostle-Cázares, B., Maldonado-Mendoza, I.E., Carrillo-González, R., Hernández-Hernández, J., Garduño, M.D., López-Meyer, M., Gómez-Flores, L., González-Chávez, M.C. (2010). Plant and fungal biodiversity from metal mine wastes under remediation at Zimapan, Hidalgo, Mexico. Environ. Pollut. 158, 1922–1931.

Trouvelot, A., Kough, J.L., Gianinazzi-Pearson, V. (1986). Mesure du taux de mycorhization d’un système radiculaire. Recherche de méthodes d’estimation ayant une signification fonctionnelle, In Physiological and genetical aspects of mycorrhizae, Gianinazzi-Pearson, V., Gianinazzi, S., Eds. INRA Presse, Paris, pp. 217-221.

**Fig. S3: Geographic location of the study sites and representative views.** A) Geographic location of the study sites (Huana and Tambillos tailings) in the Coquimbo region of northern-central Chile. B) Representative pictures of the tailings and their vegetation, including *Baccharis linearis* specimens.

**Table S1: Macronutrient and elemental characterization of substrates.**

|  |  | **Substrates^†^** |  |  |
| --- | --- | --- | --- | --- |
| **Substrate parameters**^ǂ^**^,^** ^¶^ | **TT** | **HT** | **Surrounding soil** | **FT** |
| **Organic matter (%)** | 0.32 ± 0.11**^↓↓^** | 0.16 ± 0.07**^↓↓^** | 0.31 ± 0.10**^↓↓^** | 1.07 ± 0.39**^↓↓^** |
| **Available N (mg kg^-1^)** | 4.55 ± 2.19**^↓↓^** | 10.82 ± 5.39**^↓^** | 19.56 ± 2.54 | 8.56 ± 5.95**^↓↓^** |
| **Available P (mg kg^-1^)** | 4.12 ± 1.23**^↓↓^** | 0.75 ± 0.46**^↓↓^** | 6.31 ± 5.15**^↓^** | 3.50 ± 1.47**^↓↓^** |
| **Exchangeable**  **cations (mg kg^-1^)** |  |  |  |  |
| **Na^+^** | 50.3 ± 31.7 | 208.4 ± 222.5**^↑↑^** | 86.0 ± 46.4**^↑^** | 208.8 ± 83.7**^↑↑^** |
| **Ca^2+^** | 10428 ± 3455**^↑↑^** | 11663 ± 2212**^↑↑^** | 12351 ± 4829**^↑↑^** | 8819 ± 2503**^↑↑^** |
| **Mg^2+^** | 132.8 ± 77.4 | 262.3 ± 97.1**^↑^** | 804.9 ± 257.4**^↑↑^** | 276.6 ± 116.8**^↑^** |
| **K^+^** | 91.1 ± 24.8 | 236.1 ± 110.2**^↑^** | 201.0 ± 127.1**^↑^** | 104.6 ± 41.5 |

**^†^**TT: Tambillos tailings; HT: Huana tailings; FT: Fresh Tambillos tailings.

^ǂ^Values are given as mean and standard error (2-6 replicates).

^¶^ (**↓**) Low, (**↓↓**) very low, (**↑**) high, or (**↑↑**) very high, according to standard analytical soil parameters.

*Characterization of rhizosphere substrates and fresh tailings*

Sample preparation, organic matter content and available nitrogen and phosphorous determinations were carried out as indicated in Table SI. A saturated water extract of each sample was prepared for determination of exchangeable cations.

**Table S2: Alpha diversity indices for operational taxonomic units in non-rhizosphere and rhizosphere of *Baccharis linearis* from Huana and Tambillos tailings and a surrounding soil**.

| Indices**^†^** | Sites | Root compartments | | | *p* values ^ǂ^ | | |  |
| --- | --- | --- | --- | --- | --- | --- | --- | --- |
|  |  | Rhizoplane | Rhizosphere | Non-rhizosphere | Localization | Root  Compartment | Localization  vs. Root  Compartment | |
| Richness *(S´)* | All sites | 19.40 ± 7.80 | 35.80 ± 7.73 | 36.50 ± 3.70 | 0.3824 | *** | 0.0926 | |
|  | Huana | 17.75 ± 10.04 | 40.00 ± 2.82 | 32.50 ± 5.00 |  |  |  |  |
|  | Tambillos | 26.50 ± 8.35 | 32.50 ± 10.24 | 39.50 ± 3.11 |  |  |  |  |
|  | Surrounding soil | 14.00 ± 4.90 | 35.00 ± 10.13 | 37.50 ± 3.00 |  |  |  |  |
|  | |  |  |  |  |  |  | |
| Diversity (*H´*) | All sites | 2.06 ± 0.61 | 2.94 ± 0.49 | 3.20 ± 0.20 | 0.2080 | *** | 0.2510 | |
|  | Huana | 1.97 ± 0.66 | 3.35 ± 0.07 | 3.03 ± 0.33 |  |  |  |  |
|  | Tambillos | 2.54 ± 0.78 | 2.97 ± 0.41 | 3.33 ± 0.15 |  |  |  |  |
|  | Surrounding soil | 1.67 ± 0.40 | 2.72 ± 0.99 | 3.26 ± 0.11 |  |  |  |  |
|  | |  |  |  |  |  |  | |
| Evenness *(J´)* | All sites | 0.71 ± 0.10 | 0.84 ± 0.10 | 0.89 ± 0.04 | 0.1659 | *** | 0.4728 | |
|  | Huana | 0.72 ± 0.05 | 0.91 ± 0.02 | 0.87 ± 0.06 |  |  |  |  |
|  | Tambillos | 0.77 ± 0.18 | 0.86 ± 0.05 | 0.91 ± 0.04 |  |  |  |  |
|  | Surrounding soil | 0.64 ± 0.08 | 0.76 ± 0.23 | 0.90 ± 0.02 |  |  |  |  |

**^†^**Indices values are mean and standard error from four replicates. ^ǂ^*p* values for differences in bacterial diversity indices were determined using two-way analysis of variance (ANOVA). *Statistical significance.

*Alpha diversity analysis:* Alpha diversity has been defined as an index of local diversity, which corresponds to the sum of samples richness in a grid of sampling points in a specific moment of time (Whittaker, 1972). Alpha diversity was determined through calculation of S, which in this case corresponded to the total number of T-RFs; H calculated using the Shannon–Weaver formula *H* = −Σ 𝑝i ∗ ln(𝑝i) (Blackwood et al., 2007), where 𝑝i is the proportion of an individual T-RF area relative to the sum of all T-RFs areas; and evenness (J) values were calculated with the formula 𝐽 = 𝐻 ln(𝑆)^-1^ (Pielou, 1966). For each site (soil, tailings), one-way ANOVA statistical test was carried out. To improve data interpretation, spatial groups were defined as substrate type (HT, TT, FT, surrounding soil) and root compartment (rhizoplane, rhizosphere and non-rhizosphere).

Blackwood, C.B., Hudleston, D., Zak, D.R., Buyer, J.S. (2007). Interpreting ecological diversity indices applied to terminal restriction fragment length polymorphism data: insights from simulated microbial communities. Appl. Environ. Microbiol. 73, 5276-5283.

Pielou, E.C. (1966). The measurement of diversity in different types of biological collections. J. Theor. Biol. 13, 131-144.

Whittaker, R.H. (1972). Evolution and measurement of species diversity. Taxon 21, 213- 251.

**Table S3: Alpha diversity indices for fungal operational taxonomic units (OTUs) detected in the non-rhizosphere and the rhizosphere of *Baccharis linearis* plants growing on abandoned tailing dumps (Huana and Tambillos) and a surrounding soil.**

| Indices**^†^** | Sites | Root compartments | | | *p* values ^ǂ^ | | |
| --- | --- | --- | --- | --- | --- | --- | --- |
|  |  | Rhizoplane | Rhizosphere | Non-rhizosphere | Localization | Root Compartment | Localization  vs. Root Compartment |
| Richness *(S´)* | All sites | 22.58 ± 4.76 | 22.66 ± 3.42 | 29.83 ± 5.75 | *** | 0.3511 | 0.2294 |
|  | Huana | 14.75 ± 0.50 | 18.00 ± 2.31 | 32.50 ± 5.00 |  |  |  |
|  | Tambillos | 29.25 ± 10.40 | 21.75 ± 3.30 | 27.00 ± 10.42 |  |  |  |
|  | Surrounding soil | 23.75 ± 3.40 | 28.25 ± 4.64 | 30.00 ± 1.82 |  |  |  |
|  | |  |  |  |  |  |  |
| Diversity (*H´*) | All sites | 2.46 ± 0.29 | 2.58 ± 0.29 | 2.59 ± 0.32 | ** | 0.6112 | 0.2702 |
|  | Huana | 2.22 ± 0.12 | 2.40± 0.19 | 2.33 ± 0.23 |  |  |  |
|  | Tambillos | 2.73 ± 0.39 | 2.53± 0.33 | 2.48 ± 0.62 |  |  |  |
|  | Surrounding soil | 2.44 ± 0.37 | 2.82 ± 0.37 | 2.96 ± 0.11 |  |  |  |
|  | |  |  |  |  |  |  |
| Evenness *(J´)* | All sites | 0.80 ± 0.06 | 0.83 ± 0.06 | 0.80 ± 0.07 | 0.5990 | 0.5070 | 0.1870 |
|  | Huana | 0.83 ± 0.05 | 0.83 ± 0.05 | 0.78 ± 0.10 |  |  |  |
|  | Tambillos | 0.82 ± 0.04 | 0.82 ± 0.07 | 0.76 ± 0.10 |  |  |  |
|  | Surrounding soil | 0.76 ± 0.08 | 0.84 ± 0.07 | 0.87 ± 0.02 |  |  |  |

**^†^**Indices values are given as mean and standard error from four replicates.

^ǂ^*p* values for differences in fungal diversity indices determined using two-way analysis of variance (ANOVA).

*Statistical significance.

*Alpha diversity analysis*

See footnote text in Table SIII.

**Table S4: Nutritional characterization of irradiated and non-irradiated substrates**

| **Substrate parameters** |  |  | **Substrates** |  |  |  |
| --- | --- | --- | --- | --- | --- | --- |
|  | **Tambillos** | **Irradiated Tambillos** | **Huana** | **Irradiated Huana** | **Surrounding soil** | **Irradiated surrounding soil** |
| **Organic matter (%)** | 0.22 | ~0 | 0.22 | ~0 | 0.29 | ~0 |
| **CEC mEq 100 g^-1, †^** | 22.36 | 23.84 | 28.44 | 27.96 | 31.14 | 33.32 |
| **EC mS cm^-1^** ^‡^ | 2.84 | 2.89 | 3.46 | 3.56 | 2.39 | 2.58 |
| **Available N (mg kg^-1^)** | 8.39 | <1.00 | 15.34 | <1.00 | 17.02 | <1.00 |
| **Available P (mg kg^-1^)** | 5.44 | 6.05 | 1.16 | 2.55 | 10.78 | 11.51 |
| **Available K (mg kg^-1^)** | 86.8 | 81.1 | 261.7 | 233.0 | 196.4 | 197.1 |

**^†^** CEC: Cation Exchange Capacity ^‡^ EC: Electrical Conductivity.

*Nutritional characterization of irradiated and non-irradiated substrates*

Sample preparation was carried out according to standard procedures described in ISO (1994) and USDA (1996). All samples were dried in an airflow chamber at 30ºC until a constant weight. Dry samples were passed through a 2 mm pore size sieve prior to analyses. Organic matter content was determined through the wet combustion method by Walkie-Black (Nelson and Sommers, 1982). A saturated water extract of each sample was prepared for determination of exchangeable cation contents (calcium, magnesium, sodium and potassium). The exchangeable cations were extracted with 1 M ammonium acetate at pH 7, and measured by atomic absorption spectrophotometry (Ca and Mg), and by flame photometry (Na and K) (USDA, 1996). Available nitrogen was measured by extraction with 2 M KCl and direct determination by distillation and titration, while available phosphorous and potassium were measured by extraction with 0.5 M NaHCO_3_ and spectrophotometric determination (SSSA, 1996).

ISO. (1994). Soil quality: pretreatment of samples for physico-chemical analysis. ISO. 11464.

Nelson, D.W., Sommers, L.E., 1982. Total carbon, organic carbon, and organic matter Methods of soil analysis II. American Society of Agronomy: Madison. USA. pp. 539–579.

SSSA (1996). Methods of Soil Analysis. Part 3- Chemical Methods. Soil Science Society Of America, Inc.

USDA. (1996). Soil Survey Laboratory Methods Manual, Soil Survey Investigations Report Nº 42, version 3.0, National Soil Survey Center, Natural Resources Conservation Service, United States Department of Agriculture.


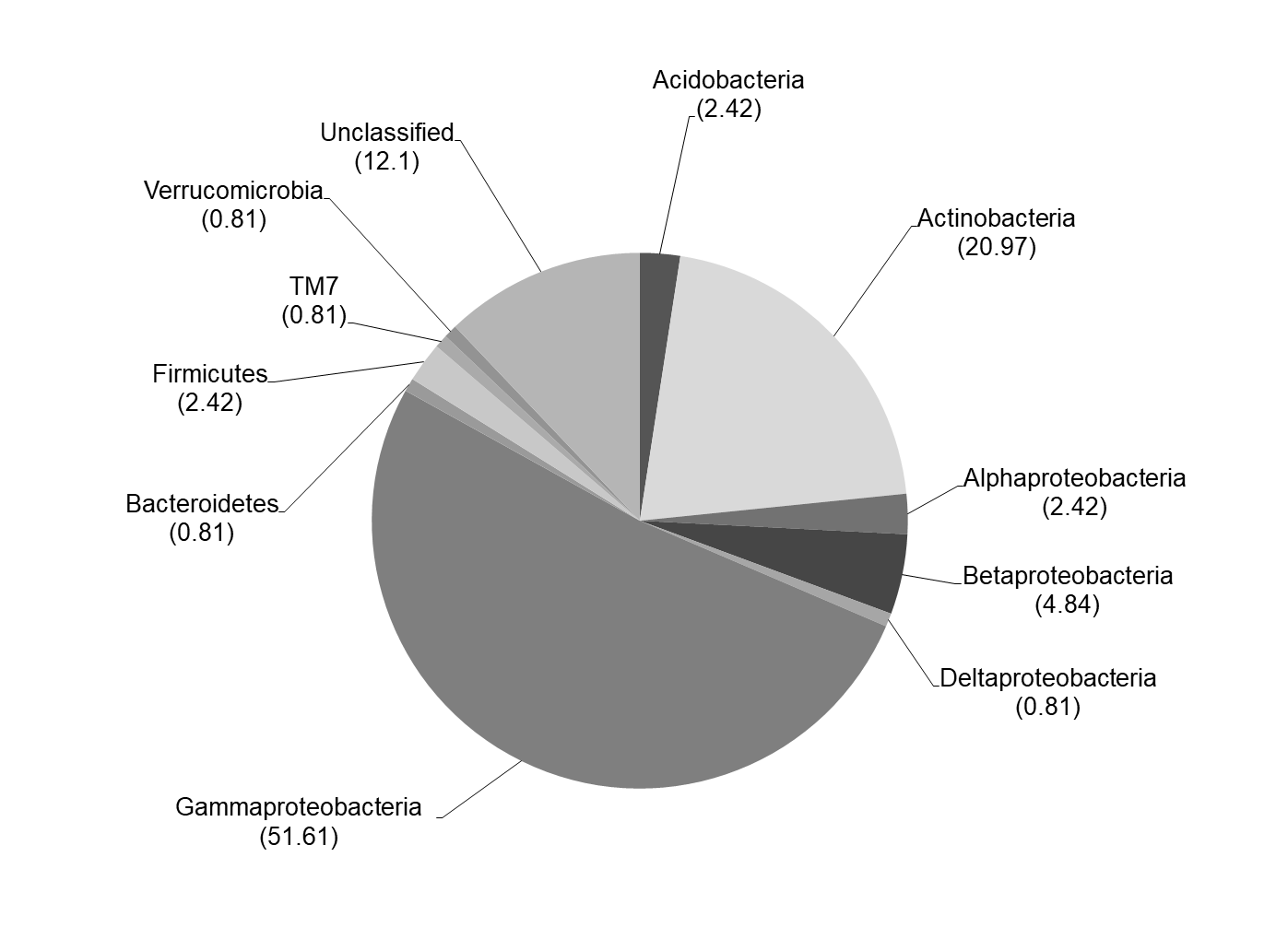


**Fig. S1: More abundant bacterial taxa from the rhizoplane of *Baccharis linearis* based on 16S rRNA clone libraries sequences.**


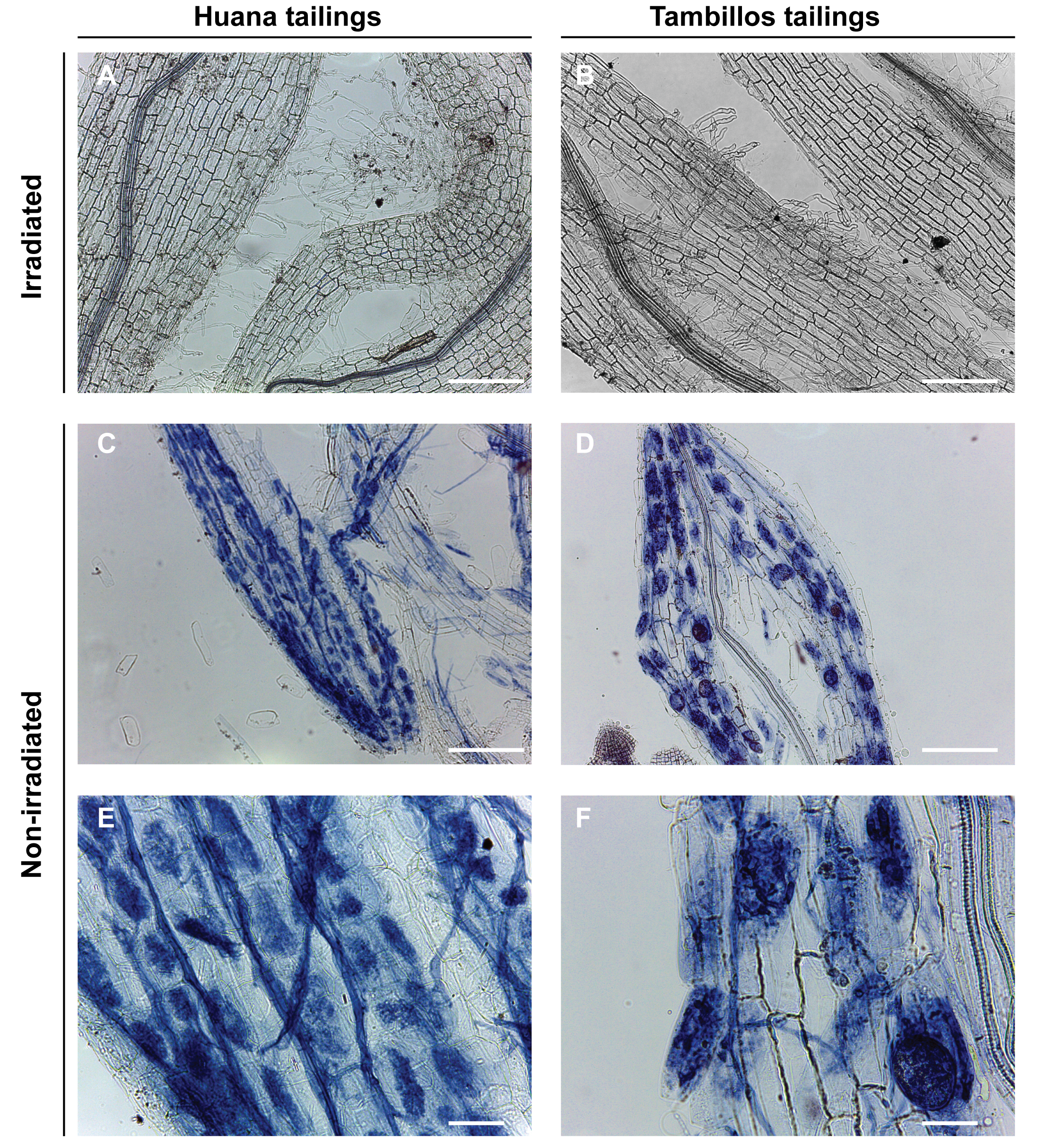


**Fig. S2: Detection of arbuscular mycorrhizal (AM) fungi in *Baccharis linearis* roots grown in non-irradiated or irradiated tailings-derived substrates.**


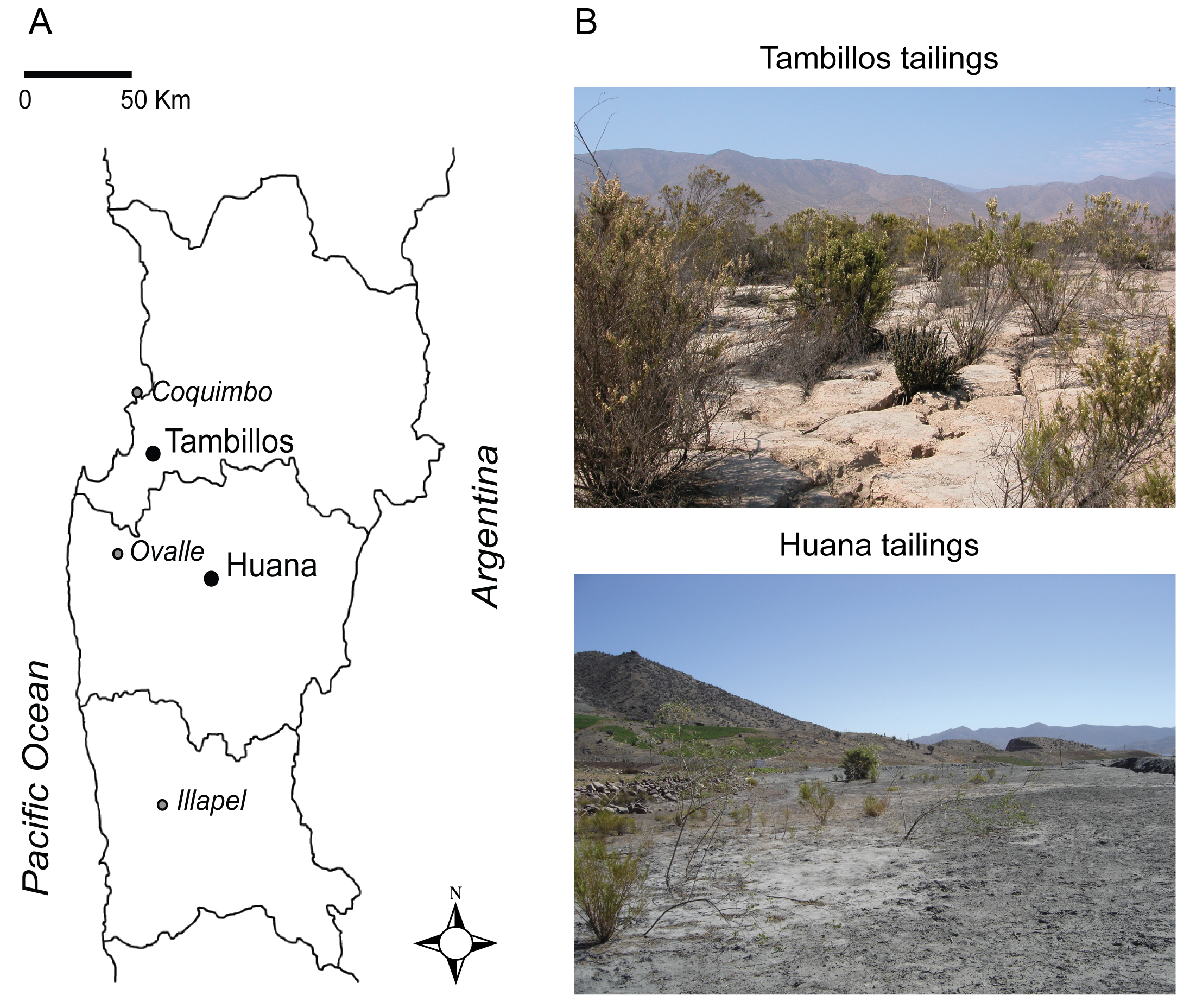


**Fig. S3: Geographic location of the study sites and representative views.** A) Geographic location of the study sites (Huana and Tambillos tailings) in the Coquimbo region of northern-central Chile. B) Representative pictures of the tailings and their vegetation, including *Baccharis linearis* specimens.
